# Human CARMIL2 deficiency underlies a broader immunological and clinical phenotype than CD28 deficiency

**DOI:** 10.1084/jem.20220275

**Published:** 2022-12-14

**Authors:** Romain Lévy, Florian Gothe, Mana Momenilandi, Thomas Magg, Marie Materna, Philipp Peters, Johannes Raedler, Quentin Philippot, Anita Lena Rack-Hoch, David Langlais, Mathieu Bourgey, Anna-Lisa Lanz, Masato Ogishi, Jérémie Rosain, Emmanuel Martin, Sylvain Latour, Natasha Vladikine, Marco Distefano, Taushif Khan, Franck Rapaport, Marian S. Schulz, Ursula Holzer, Anders Fasth, Georgios Sogkas, Carsten Speckmann, Arianna Troilo, Venetia Bigley, Anna Roppelt, Yael Dinur-Schejter, Ori Toker, Karen Helene Bronken Martinsen, Roya Sherkat, Ido Somekh, Raz Somech, Dror S. Shouval, Jörn-Sven Kühl, Winnie Ip, Elizabeth M. McDermott, Lucy Cliffe, Ahmet Ozen, Safa Baris, Hemalatha G. Rangarajan, Emmanuelle Jouanguy, Anne Puel, Jacinta Bustamante, Marie-Alexandra Alyanakian, Mathieu Fusaro, Yi Wang, Xiao-Fei Kong, Aurélie Cobat, David Boutboul, Martin Castelle, Claire Aguilar, Olivier Hermine, Morgane Cheminant, Felipe Suarez, Alisan Yildiran, Aziz Bousfiha, Hamoud Al-Mousa, Fahad Alsohime, Deniz Cagdas, Roshini S. Abraham, Alan P. Knutsen, Borre Fevang, Sagar Bhattad, Ayca Kiykim, Baran Erman, Tugba Arikoglu, Ekrem Unal, Ashish Kumar, Christoph B. Geier, Ulrich Baumann, Bénédicte Neven, CARMIL2 Consortium, Julie Calas, Elizabeth Feuille, Angela Chan, Gozde Yesil, Justine Nammour, Élise Bandet, Capucine Picard, Ibtihal Benhsaien, Peter Lang, Faranaz Atschekzei, Klaus Warnatz, Sophie Hambleton, Mukesh Desai, Elif Karakoc-Aydiner, Burcu Kolukisa, Saleh Al-Muhsen, Mohammed F. Alosaimi, Funda Cipe, Anas M. Alazami, Gonca Hancioglu, Bilge Can Meydan, Hanne S. Sorte, Asbjørg Stray-Pedersen, Geetha Mammayil, Nazan Tökmeci, Anna Shcherbina, Polina Stepensky, Adeeb NaserEddin, Claire Rouzaud, Akihiro Hoshino, Oded Shamriz, Oren Ledder, Maria Elena Maccari, Carla N. Castro, Bodo Grimbacher, Reinhold E. Schmidt, Matthew Collin, Victorya Zakharova, Meino Rohlfs, Christoph Walz, Laurent Abel, Bernard Malissen, Nico Marr, Christoph Klein, Jean-Laurent Casanova, Fabian Hauck, Vivien Béziat

**Affiliations:** 1 Laboratory of Human Genetics of Infectious Diseases, Necker Branch, INSERM, Necker Hospital for Sick Children, Paris, France; 2 Imagine Institute, University of Paris-Cité, Paris, France; 3 Pediatric Immunology-Hematology and Rheumatology Unit, Necker Hospital for Sick Children, AP-HP, Paris, France; 4 St. Giles Laboratory of Human Genetics of Infectious Diseases, Rockefeller Branch, The Rockefeller University, New York, NY; 5 Dept. of Pediatrics, Dr. von Hauner Children’s Hospital, University Hospital, Ludwig-Maximilians-Universität Munich, Munich, Germany; 6 Dept. of Human Genetics, McGill University, Montreal, Quebec, Canada; 7 Laboratory of Lymphocyte Activation and Susceptibility to EBV infection, INSERM UMR 1163, Paris, France; 8 Research Branch, Sidra Medicine, Doha, Qatar; 9 Dept. of Women and Child Health, Hospital for Children and Adolescents, Hospitals University of Leipzig, Leipzig, Germany; 10 Children’s Hospital, University of Tübingen, Tübingen, Germany; 11 Dept. of Pediatrics, Institute of Clinical Sciences, University of Gothenburg, Gothenburg, Sweden; 12 The Queen Silvia Children’s Hospital, Gothenburg, Sweden; 13 Dept. of Immunology and Rheumatology, Medical School Hannover, Hanover, Germany; 14 Dept. of Pediatrics and Adolescent Medicine, Division of Pediatric Hematology and Oncology and Center for Chronic Immunodeficiency (CCI), Institute for Immunodeficiency, Medical Center, Faculty of Medicine, University of Freiburg, Freiburg, Germany; 15 Dept. of Rheumatology and CCI for Chronic Immunodeficiency, Division of Immunodeficiency, Medical Center, Faculty of Medicine, University of Freiburg, Freiburg, Germany; 16 Translational and Clinical Research Institute and NIHR Newcastle Biomedical Research Centre, Newcastle University and Newcastle upon Tyne Hospitals NHS Foundation Trust, Newcastle upon Tyne, UK; 17 Dept. of Immunology, Dmitry Rogachev National Medical Research Center of Pediatric Hematology, Oncology and Immunology, Moscow, Russia; 18 Dept. of Bone Marrow Transplantation, Hadassah Medical Center, Faculty of Medicine, Hebrew University, Jerusalem, Israel; 19 Faculty of Medicine, Hebrew University of Jerusalem, The Allergy and Clinical Immunology Unit, Shaare Zedek Medical Center, Jerusalem, Israel; 20 Dept. of Pediatric Immunology, Rikshospitalet, Oslo University Hospital, Oslo, Norway; 21 Acquired Immunodeficiency Research Center, Isfahan University of Medical Sciences, Isfahan, Iran; 22 Dept. of Pediatric Hematology/Oncology, Schneider Children’s Medical Center of Israel, Petah Tikva, Israel; 23 The Institute of Gastroenterology, Nutrition and Liver diseases, Schneider Children's Medical Center of Israel, Petah Tikva, Israel, and The Sackler Faculty of Medicine, Tel Aviv University, Tel Aviv, Israel; 24 The Sackler Faculty of Medicine, Tel Aviv University, Tel Aviv Israel; The Institute of Gastroenterology, Nutrition and Liver Diseases, Schneider Children's Hospital, Petach-Tikva, Israel; Ben-Gurion University of the Negev, Beer Sheva, Israel; 25 Dept. of Immunology, Great Ormond Street Hospital, London, UK; 26 Dept. of Pediatrics, Nottingham University Hospitals NHS Trust, Nottingham, UK; 27 Dept. of Pediatric Allergy and Immunology, Marmara University, Istanbul, Turkey; 28 Division of Hematology, Oncology and Bone Marrow Transplant, Dept. of Pediatrics, Nationwide Children’s Hospital, Columbus, OH; 29 Center for the Study of Primary Immunodeficiencies, Necker Hospital for Sick Children, Paris, France; 30 Dept. of Immunology, Necker Hospital for Sick Children, AP-HP, Paris, France; 31 Dept. of Clinical Immunology, AP-HP, Saint-Louis Hospital, Paris, France; 32 Necker Pasteur Center for Infectious Diseases and Tropical Medicine, Necker Hospital for Sick Children, AP-HP, Paris, France; 33 Dept. of Clinical Hematology, Necker Hospital for Sick Children, AP-HP, Paris, France; 34 Dept. of Pediatric Immunology and Allergy, Ondokuz Mayis University Medical School, Samsun, Turkey; 35 Clinical Immunology, Inflammation and Auto-immunity Laboratory, Faculty of Medicine and Pharmacy of Casablanca, Hassan II University, Casablanca, Morocco; 36 Translational Genomics, Centre for Genomic Medicine, King Faisal Specialist Hospital and Research Center, Riyadh, Saudi Arabia; 37 Pediatric Intensive Care Unit, Dept. of Pediatrics, King Saud University Medical City, King Saud University, Riyadh, Saudi Arabia; 38 Immunology Research Laboratory, Dept. of Pediatrics, College of Medicine, King Saud University, Riyadh, Saudi Arabia; 39 Section of Pediatric Immunology, Hacettepe University, Ihsan Dogramaci Children’s Hospital, Ankara, Turkey; 40 Dept. of Pathology and Laboratory Medicine, Nationwide Children’s Hospital, Columbus, OH; 41 Pediatric Allergy and Immunology, Cardinal Glennon Children’s Hospital, St. Louis, MO; 42 Section of Clinical Immunology and Infectious Diseases, Oslo University Hospital, Oslo, Norway; 43 Dept. of Pediatrics, Aster CMI Hospital, Bangalore, India; 44 Istanbul University-Cerrahpasa, Cerrahpasa School of Medicine, Pediatric Immunology and Allergy, Istanbul, Turkey; 45 Institute of Child Health, Hacettepe University, Ankara, Turkey; 46 Can Sucak Research Laboratory for Translational Immunology, Hacettepe University, Ankara, Turkey; 47 Dept. of Pediatrics, Division of Pediatric Allergy and Immunology, Mersin University Faculty of Medicine, Mersin, Turkey; 48 Division of Pediatric Hematology Oncology, Dept. of Pediatrics, Erciyes University Faculty of Medicine, Kayseri, Turkey; 49 Division of Bone Marrow Transplantation and Immune Deficiency, Dept. of Pediatrics, Cincinnati Children’s Hospital Medical Center, Cincinnati, OH; 50 Dept. of Paediatric Pulmonology, Allergy and Neonatology, Hannover Medical School, Hannover, Germany; 51 Institute of Pathology, Faculty of Medicine, Ludwig-Maximilians-Universität München, Munich, Germany; 52 Centre d’Immunologie de Marseille-Luminy, Aix-Marseille Université, INSERM, CNRS, Marseille, France; 53 Howard Hughes Medical Institute, New York, NY; 54 Dept. of Pediatrics, Necker Hospital for Sick Children, Paris, France

## Abstract

Patients with inherited CARMIL2 or CD28 deficiency have defective T cell CD28 signaling, but their immunological and clinical phenotypes remain largely unknown. We show that only one of three CARMIL2 isoforms is produced and functional across leukocyte subsets. Tested mutant *CARMIL2* alleles from 89 patients and 52 families impair canonical NF-κB but not AP-1 and NFAT activation in T cells stimulated via CD28. Like CD28-deficient patients, CARMIL2-deficient patients display recalcitrant warts and low blood counts of CD4^+^ and CD8^+^ memory T cells and CD4^+^ T_REG_s. Unlike CD28-deficient patients, they have low counts of NK cells and memory B cells, and their antibody responses are weak. CARMIL2 deficiency is fully penetrant by the age of 10 yr and is characterized by numerous infections, EBV^+^ smooth muscle tumors, and mucocutaneous inflammation, including inflammatory bowel disease. Patients with somatic reversions of a mutant allele in CD4^+^ T cells have milder phenotypes. Our study suggests that CARMIL2 governs immunological pathways beyond CD28.

## Teaser

Inherited CARMIL2 deficiency underlies infections, EBV^+^ smooth muscle tumors, and mucocutaneous inflammation. CARMIL2 deficiency impairs CD28 signaling only partially in T cells. The comparison of CARMIL2 and CD28 deficiency in humans suggests that CARMIL2 governs immunological pathways beyond CD28.

## Graphical Abstract

**Figure:**
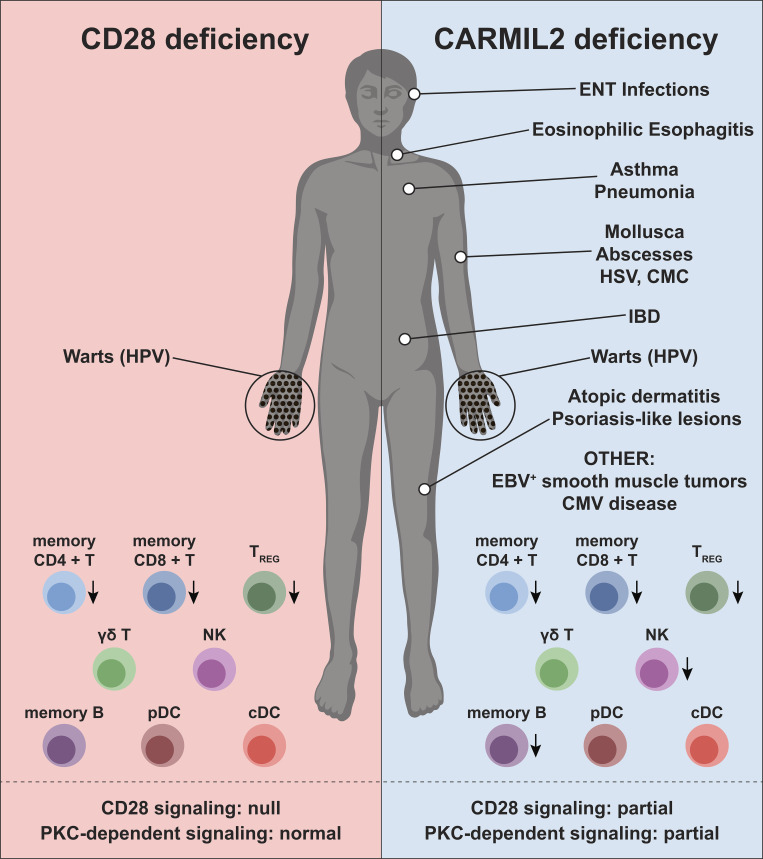

## Introduction

In the two-signal model of T cell activation, the first signal is delivered via the TCR following the recognition of antigenic peptides bound to MHC molecules. The second signal is provided by the CD28 co-stimulator, following its binding to its ligands (CD80 or CD86) on APC. After T cell activation, TCR and CD28 form microclusters that move toward the center of the immune synapse, forming a central supramolecular activation complex. Acting in synergy, the TCR and CD28 trigger the association of the cytosolic adaptor CARD11 with BCL10 and MALT1 to form the CBM (CARD11-BCL10-MALT1) complex, which stimulates NF-κB signaling (Thome et al., 2010; Jiang and Lin, 2012; Wang et al., 2012). In murine T cells, capping protein regulator and myosin 1 linker 2 (CARMIL2), previously known as RLTPR (RGD, leucine-rich repeat [LRR], tropomodulin, and proline-rich-containing protein), has been shown to be an essential scaffolding protein for CD28 costimulation (Liang et al., 2013). CARMIL2 interacts with CARD11 (Roncagalli et al., 2016), and, in T cells expressing a mutated CARMIL2 allele, the accumulation of CARD11 to the central supramolecular activation complex and NF-κB activation are abolished (Liang et al., 2013). In mice, CARMIL2 is also essential for the development of regulatory T cells (T_REG_s; Liang et al., 2013), and the in vitro differentiation of type 1 helper T cells (T_H1_) and T_H17_ cells, whereas it is redundant for T_H2_ differentiation (Roncagalli et al., 2016). Despite its expression by murine B cells, CARMIL2 deficiency affects only murine responses to T cell–dependent antigens, with T cell–independent responses remaining intact (Roncagalli et al., 2016). Finally, murine CARMIL2 is expressed in natural killer (NK) cells and plasmacytoid dendritic cells (pDCs), but its function in these cells remains unknown (Roncagalli et al., 2016).

In humans, biallelic *CARMIL2* loss-of-function (LOF) variants cause a combined immunodeficiency, with susceptibility to viral, bacterial, mycobacterial, and fungal infections, immune dysregulation in the gut and skin (Schober et al., 2017; Wang et al., 2016; Magg et al., 2019), and a particular susceptibility to EBV^+^ smooth muscle tumors (EBV^+^ SMTs; Schober et al., 2017; Magg et al., 2018). Affected individuals have abnormally low proportions of memory CD4^+^ T cells, T_REG_s, and memory B cells (Wang et al., 2016). As in mice, mutant human T cells display impairments of CD28 signaling, T_H1_ and T_H17_ cell differentiation in vitro, an abnormal cytoskeletal organization interfering with T cell polarity and migration, and impaired B cell responses in vivo (Wang et al., 2016; Schober et al., 2017). The recent discovery of individuals with inherited biallelic CD28 deficiency has challenged our understanding of the role of CARMIL2 (Béziat et al., 2021). Studies of human CD28 deficiency have revealed that CD28 signaling is required for immunity to ⍺- and γ-papillomaviruses (HPV) but otherwise largely redundant (Béziat et al., 2021). In turn, this suggested that impaired CD28 activation could account for susceptibility to HPV in CARMIL2-deficient individuals. Conversely, the apparently more severe and broader clinical phenotype of individuals with CARMIL2 deficiency than of those with CD28 deficiency suggests an involvement of CARMIL2 in additional signaling pathways. Consistent with this hypothesis, we previously reported an impairment of NF-κB activation downstream from surface IgM in CARMIL2-deficient B cells (Wang et al., 2016). However, we were unable to rescue any T or B cell phenotype in human cells with a WT copy of the “canonical” isoform of *CARMIL2* (Wang et al., 2016; Schober et al., 2017). Moreover, the clinical phenotypes of CARMIL2 and CD28 deficiencies have been determined from only small numbers of cases. It is, therefore, important to undertake an in-depth characterization of the genetic, immunological, and clinical features of inherited CARMIL2 deficiency, to set the stage for CARMIL2-signaling studies in humans.

## Results

### Only the *CARMIL2* isoform 3 is expressed in human leukocyte subsets

**Figure 1.**
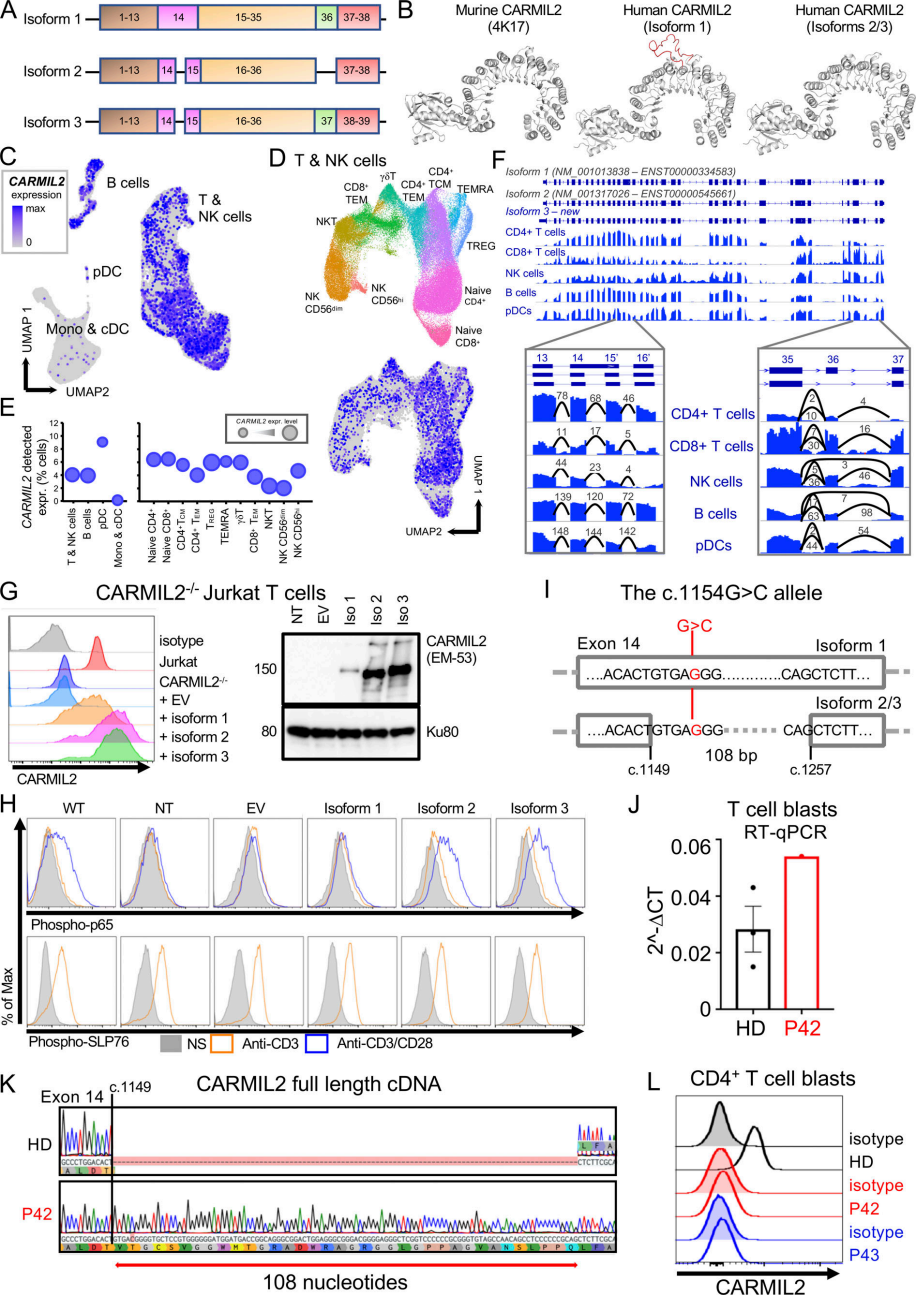
**Genetic analysis demonstrates that the *CARMIL2* isoform 3 is canonical and that the isoform 1 is pathogenic. (A)** Schematic representation of the three *CARMIL2* isoforms. Isoform 1 (ENST00000334583.11, transcript 1) encodes a 1435-amino acid protein and has 38 exons. Isoform 2 (ENST00000545661.5) encodes a 1372-amino acid protein and has 38 exons. Isoform 2 lacks 108 nucleotides present in isoform 1 due to an additional intron within exon 14. It also loses the whole of exon 36 but retains the same open reading frame. Isoform 3 (not reported in the Ensembl database) encodes a 1399-amino acid protein and has 39 exons. Relative to isoform 1, isoform 3 loses part of exon 14, but retains exon 36. **(B)** *CARMIL2* murine and human isoform 1-3 crystal structures. **(C)** scRNA-seq UMAP clustering of PBMCs from 6 HDs showing normalized *CARMIL2* expression in major cell lineages. **(D)** Reclustering of the NK and T cell cluster from C identifying the different cellular subsets, with superimposed CARMIL2 levels. **(E)** Bubble graph presenting the percentage of cells for which *CARMIL2* transcripts are detected by scRNA-seq for each cluster presented in C and D. Bubble size indicates median relative expression for CARMIL2-positive cells. **(F)** Genome browser snapshot showing bulk RNA-seq coverage for sequence reads aligned to the *CARMIL2* gene. The structure of the two known isoforms is shown at the top; blue rectangles represent exons and connecting lines represent introns. The structure of isoform 3 is also shown; this was the predominant isoform detected in the cells analyzed. The insets show sequence coverage and the number of detected spliced reads for exons 13 to 16 and exons 35 to 37. Monos, monocytes. **(G)** CARMIL2 expression, assessed by FACS (*N* = 3) and Western blotting (*N* = 2) in a CARMIL2-deficient Jurkat T cell line, after transduction with an empty lentivirus, or lentivirus encoding each of the three CARMIL2 isoforms. **(H)** Phospho-p65 and phospho-SLP76 levels in cells, as described in G, following stimulation with anti-CD3, or anti-CD3/CD28 mAb. (*N* = 3). **(I)** Schematic representation of the *CARMIL2* gene isoform 1 (ENST00000334583.11), isoform 2 (ENST00000545661.5), and isoform 3, for exon 14. The G>C substitution is indicated in position c.1154 for isoform 1, and c.1149 + 5 for isoforms 2 and 3. **(J)** Total mRNA was extracted from the T cell blasts of a HD and P42 (c.1149 + 5G>C). Total mRNA was subjected to RT-qPCR for the assessment of total *CARMIL2* expression. Data are displayed as 2^−ΔCt^ values after normalization against endogenous *GUS* control gene expression. Mean ± SEM of three technical replicates (*N* = 2). **(K)** PCR amplification of the full-length *CARMIL2* cDNA from T cell blasts from a HD and P42. Electropherograms show an insertion of 108 nucleotides into exon 14 of the patient’s sequence. The predicted impact on the translated protein in P42 corresponds to the exact sequence of isoform 1. **(L)** CARMIL2 protein levels in T cell blasts, as assessed by intracellular FACS on CD4^+^ T cells for a HD, P42 and P43, both homozygous for the G>C substitution shown in I. Source data are available for this figure: SourceData F1.

Two *CARMIL2* transcripts arising from alternative splicing are described as protein-coding in the Ensembl database (Fig. 1 A). The first (ENST00000334583.11; transcript 1) encodes a 1435-amino acid protein with 38 exons (isoform 1). The second (ENST00000545661.5; transcript 2) encodes a 1372-amino acid protein with 38 exons (isoform 2). Transcript 2 has 108 nucleotides fewer than transcript 1 due to the presence of an additional intron within exon 14, and the loss of exon 36, but it retains the same open reading frame. mRNA sequencing in adult T cell leukemia/lymphoma, cutaneous cytotoxic T cell lymphoma, and CD4^+^ primary human T cells has revealed a third transcript (transcript 3) not reported in Ensembl (Park et al., 2017; Uchida et al., 2021), encoding a 1399-amino acid protein with 39 exons (isoform 3). Transcript 3 also lacks part of exon 14, but it retains exon 36. The retention of part of exon 14 in isoform 1 is predicted to result in an additional loop projecting outside the CARMIL2 LRR crystal structure (Fig. 1 B). We assessed the differential expression of *CARMIL2* transcripts in various leukocyte subsets, by analyzing 3′ single-cell (sc) RNA sequencing (RNA-seq) data from healthy control primary peripheral blood mononuclear cells (PBMCs). Consistent with the levels of CARMIL2 protein measured by FACS (Wang et al., 2016), we found high levels of *CARMIL2* mRNA in all T cell subsets, B cells, NK cells, and pDCs, but weak expression in conventional dendritic cells (cDCs) and monocytes (Fig. 1, C−E). For resolution of the entire coding sequence of *CARMIL2*, we analyzed total RNA-seq data for healthy control primary CD4^+^ and CD8^+^ T cells, B cells, NK cells, and pDCs. In all leukocyte subsets, part of exon 14 was spliced out and exon 36 was retained; by contrast, a minority of transcripts in NK and B cells spliced out exon 36, (Fig. 1 F), suggesting that isoform 3 is the predominant form expressed in all human immune cells.

### *CARMIL2* isoform 1 is non-functional in human leukocytes

We investigated the function of each of the *CARMIL2* transcripts, using a lentiviral system to rescue CARMIL2-deficient Jurkat cells with a cDNA encoding isoform 1, 2, or 3 and analyzing NF-κB activation following stimulation with anti-CD3, -CD28, or -CD3/CD28 mAb. All three isoforms were expressed in transduced Jurkat cells, but the levels of isoform 1 were lower than those of isoforms 2 and 3 (Fig. 1 G). Only isoforms 2 and 3 were able to rescue NF-κB p65 phosphorylation in response to CD28 stimulation (Fig. 1 H). These data demonstrate that isoform 1 is LOF, at least in terms of NF-κB activation downstream from CD28. We studied seven individuals from three unrelated kindreds (P41 to P44 and P79 to P81 in Table S1), bearing the same homozygous c.1154G>C substitution, predicted to result in a p.Arg385Thr missense variant located in exon 14 of isoform 1 (Fig. 1 I). This G>C substitution was predicted to affect the splicing of transcripts 2 and 3 at position c.1149 + 5 (Fig. 1 I). We studied the effect of this variant on the *CARMIL2* mRNA extracted from the T cell blasts of P42. Reverse-transcription quantitative real time-PCR (RT-qPCR) showed that *CARMIL2* mRNA levels were higher than those in healthy donor (HD) cells (Fig. 1 J). Transcript 3 was expressed in HD cells, but RT-PCR and Sanger sequencing identified only transcript 1 in P42 T cell blasts, with the retention of 108 nucleotides of intron 14 (Fig. 1 K). Furthermore, FACS did not detect endogenous CARMIL2 expression in T cell blasts from P42 and P43 (Fig. 1 L). Thus, the retention of intron 14 probably destabilizes the CARMIL2 protein, resulting in its degradation. These findings suggest that *CARMIL2* isoform 3 is the predominant product expressed and functional in human leukocytes and should, therefore, be considered the canonical isoform.

### Biallelic *CARMIL2* germline variants are pathogenic

**Figure 2.**
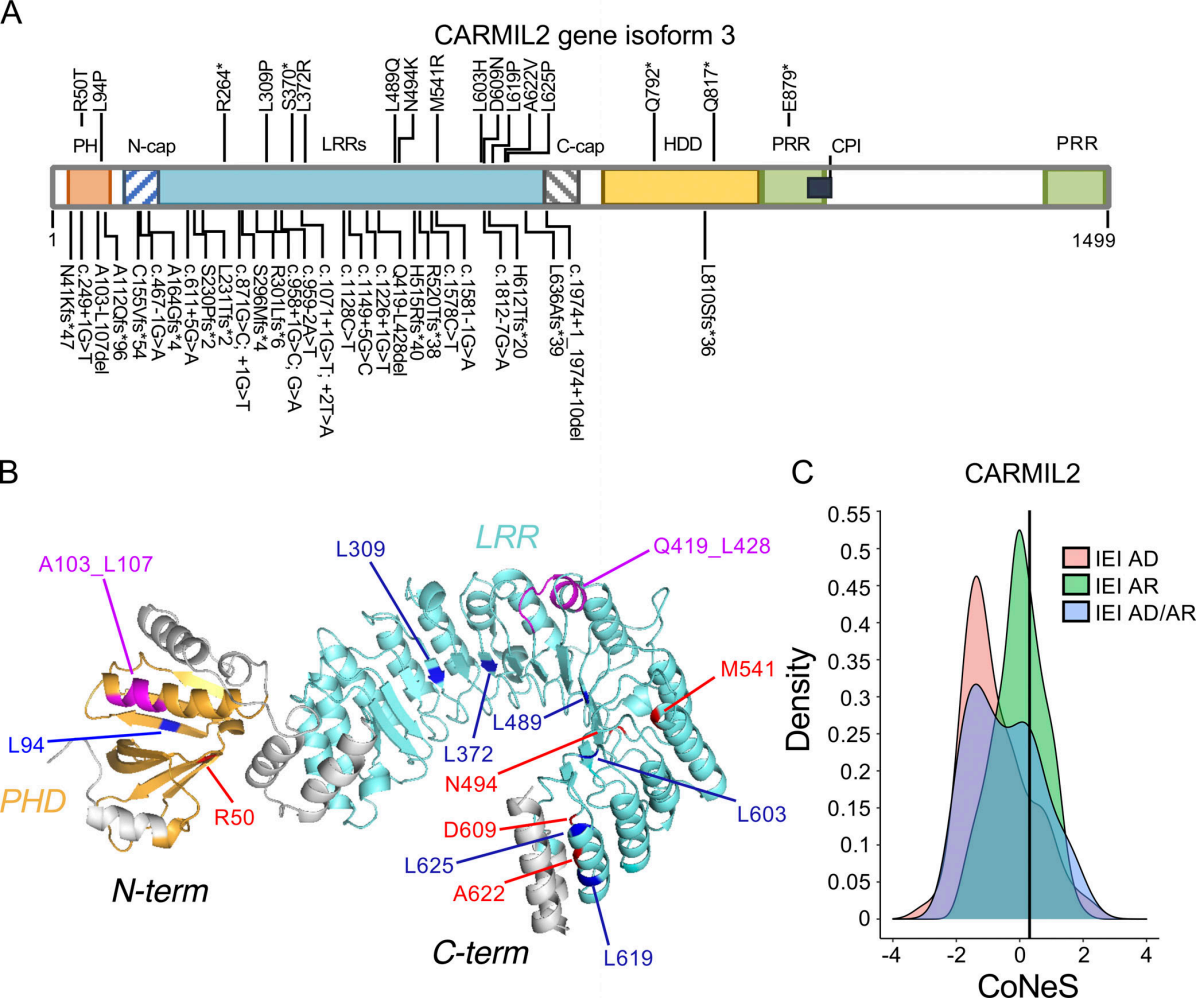
**Germline CARMIL2 mutations. (A)** Schematic representation of the *CARMIL2* gene (isoform 3), with the 49 mutations studied. Functional domains are also indicated: PH, pleckstrin homology domain; HDD, homodimerization domain; CPI, capping protein-interacting domain; PRR, proline-rich repeat domain; C-cap, C-terminal cap of the LRR; N-cap, N-terminal cap of the LRR. **(B)** Missense and in-frame deletion mutations mapped onto the crystal structure of *CARMIL2* isoform 3, modeled from murine *Rltpr* (4K17) with SWISS-MODEL (Waterhouse et al., 2018). The c.871G>C splicing variant is not shown. **(C)** Consensus negative score of *CARMIL2* and its distribution for genes causing inborn errors of immunity (IEI), according to disease mode of inheritance. AD, autosomal dominant; AR, autosomal recessive.

**Figure S1.**
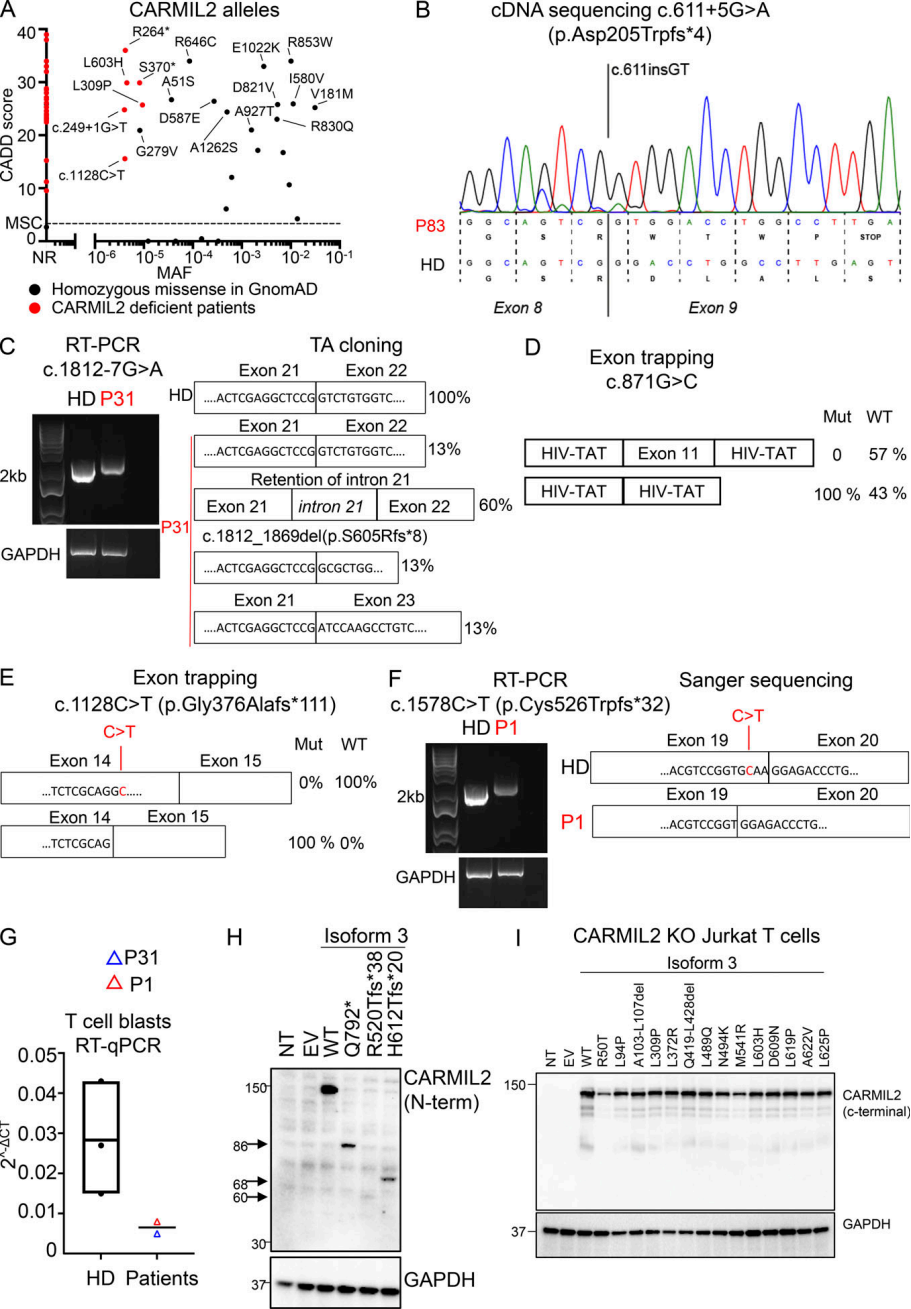
**In vitro validation of CARMIL2 variants. (A)** CADD score (y axis) plotted against MAF (x axis) for the homozygous variants present in the gnomAD database (http://gnomad.broadinstitute.org). The variants found in gnomAD and tested functionally are annotated in black, and the mutations confirmed in CARMIL2-deficient patients are annotated in red. MSC, mutation significance cutoff; NR, not reported. **(B)** Total mRNA was extracted from the T cell blasts of P83 and a HD, amplified with RT-PCR, and subjected to Sanger sequencing. **(C)** Total mRNA was extracted from the T cell blasts of a HD and P31, and subjected to RT-PCR, followed by TA cloning. PCR amplification of the *CARMIL2* cDNA is shown, with *GAPDH* cDNA as a positive control. **(D)** Exon-trapping experiments were conducted for the c.871G>C allele. Control genomic DNA was inserted into the pspl3 plasmid (WT) and subjected to site-directed mutagenesis to obtain the c.871G>C-encoding plasmid (Mut). Total mRNA was extracted from COS-7 cells transfected with the WT and Mut plasmids and subjected to RT-PCR. PCR amplification of the *CARMIL2* cDNA, with aberrant splicing detected in 100% of the screened colonies. HIV-Tat are exons from the pspl3 plasmid. *N* = 1. **(E)** Exon-trapping was performed on genomic DNA extracted from the T cell blasts of a HD and P4 (c.1128C>T) and subjected to TA cloning. Schematic representation of the PCR products, showing abnormal splicing in 100% of the screened colonies, relative to control cells. **(F)** Total mRNA was extracted from the T cell blasts of a HD and P1 (c.1578C>T) and subjected to RT-PCR. PCR amplification and Sanger sequencing of the *CARMIL2* cDNA showed an aberrant product in P1. *N* = 1. **(G)** Total mRNA was extracted from the T cell blasts of three HDs, P1, and P31. Total mRNA was subjected to RT-qPCR for the assessment of total *CARMIL2* expression. Data are displayed as 2^−ΔCt^ values after normalization according to endogenous *GUS* control gene expression. The bar represents the mean value in controls. *N* = 1. **(H)** Western blot analysis of CARMIL2 levels in total cell extracts from HEK293T cells transfected with a pcDNA3.1 plasmid, either empty (EV) or containing WT isoform 3 (WT), or mutant forms found in CARMIL2-deficient patients. Two Abs were used: an Ab against the N-terminus of CARMIL2, and an Ab against GAPDH. The data shown are representative of two independent experiments. **(I)** Western blot analysis of CARMIL2 levels in total cell extracts from non-transduced CARMIL2 KO Jurkat T cells, or after transduction with an empty lentivirus (EV), a lentivirus encoding WT isoform 3 (WT), or the missense and in-frame deletion variants identified in CARMIL2-deficient individuals (*N* = 2). Source data are available for this figure: SourceData FS1.

We established biological and clinical phenotypes of CARMIL2 deficiency by studying 89 individuals from 52 unrelated families (Table S1) originating from 23 countries. 28 families were multiplex, accounting for 65 cases. 24 cases were sporadic. 41 individuals had never been reported. We identified 49 germline *CARMIL2* variants: 13 missense, 5 nonsense, 13 frameshift, 2 in-frame deletions, 14 splice, and 2 synonymous variants (Fig. 2, A and B). Six variants were located within the pleckstrin homology domain, three were in the N-Cap domain, 35 variants were in the LRR, one was in the C-Cap domain, three were in the homodimerization domain, and one was in the proline-rich region. The p.Leu603His, p.His612Thrfs*20, c.1149 + 5G>C, and p.Leu810Serfs*36 variants were recurrent, suggesting a founder effect in the Norwegian, Saudi, Turkish, and Mexican populations, respectively (Table S1). The c.1226 + 1G>T variant was also recurrent in an Iranian and an Indian family, suggesting a hotspot or founder effect. Four individuals carried compound heterozygous variants (Table S1). All missense variants and in-frame deletions affected the amino acids present in the highly conserved pleckstrin homology (3/15) or LRR (12/15) domains (Fig, 2, A and B). 10 of the 13 missense variants and one of the two in-frame deletions affected residues conserved throughout evolution, at least as far back as *Drosophila*. The remaining variants or deletions affected residues conserved in at least all mammals. Only six variants were reported in a monoallelic state, with a minor allele frequency (MAF) below 10^−5^ (gnomAD V2.1; Fig. S1 A). All variants were predicted to be highly deleterious, with a combined annotation-dependent depletion (CADD) score above the mutation significance cutoff (Itan et al., 2016). Moreover, no homozygous predicted LOF *CARMIL2* alleles were reported in gnomAD. The consensus negative score (Rapaport et al., 2021) of this gene is 0.31, indicating that *CARMIL2* is not under strong negative selection and that the biallelic variants described here follow an autosomal recessive mode of inheritance (Fig. 2 C).

### In vitro and ex vivo validation of *CARMIL2* variant alleles

**Figure 3.**
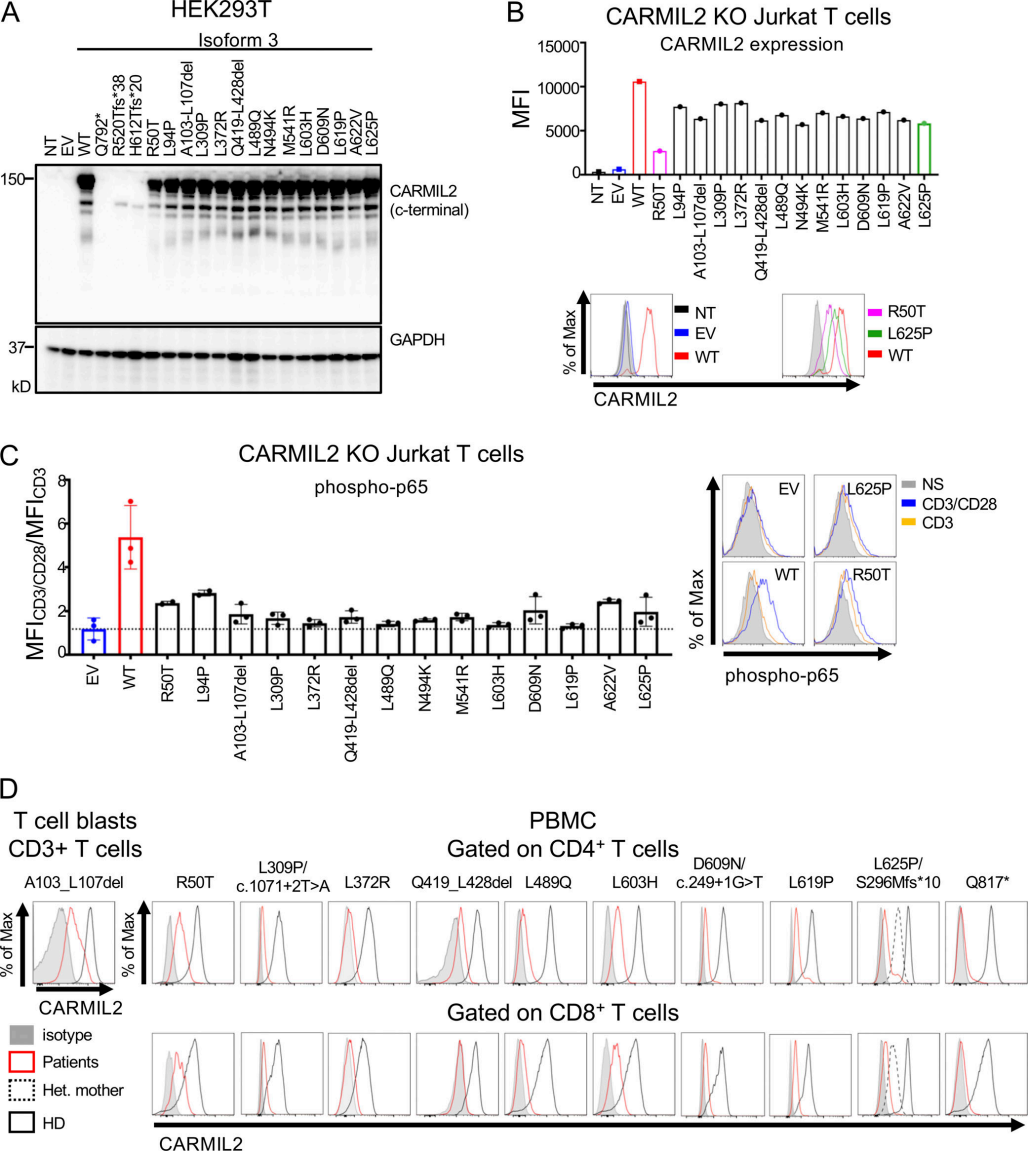
**In vitro and ex vivo validation of CARMIL2 alleles. (A)** Western blot analysis of CARMIL2 levels in total cell extracts from HEK293T cells transfected with a pcDNA3.1 plasmid, either empty (EV), or containing the WT isoform 3 (WT) or mutant forms, including all the missense and in-frame deletion variants found in CARMIL2-deficient individuals. Two Abs were used: an Ab against the C-terminus of CARMIL2 (EM-53), and an Ab against GAPDH. The data shown are representative of two independent experiments. **(B)** CARMIL2 protein levels in Jurkat T cells, as assessed by intracellular FACS, after transduction with an empty lentivirus, or a lentivirus encoding the WT isoform 3 (WT) or the missense mutants found in CARMIL2-deficient individuals. Mean fluorescence intensity (MFI; top panel) and FACS histograms (lower panel) for CARMIL2 (*N* = 2). **(C)** Phospho-p65 in Jurkat T cells, as described in B, following stimulation with anti-CD3 mAb with or without anti-CD28 mAb. The data represent the ratio of the MFI after subtraction of the MFI in the not-stimulated cells. The bar represents the mean. Error bars represent the SD. *N* = 3. **(D)** CARMIL2 protein levels, as assessed by intracellular FACS, in CD4^+^ and CD8^+^ T cells, for a representative HD, and patients homozygous for five missense mutations, compound heterozygous for three missense, frameshift and splice-site mutations, two in-frame deletion mutations, and one stop codon mutation. Source data are available for this figure: SourceData F3.

**Figure S2.**
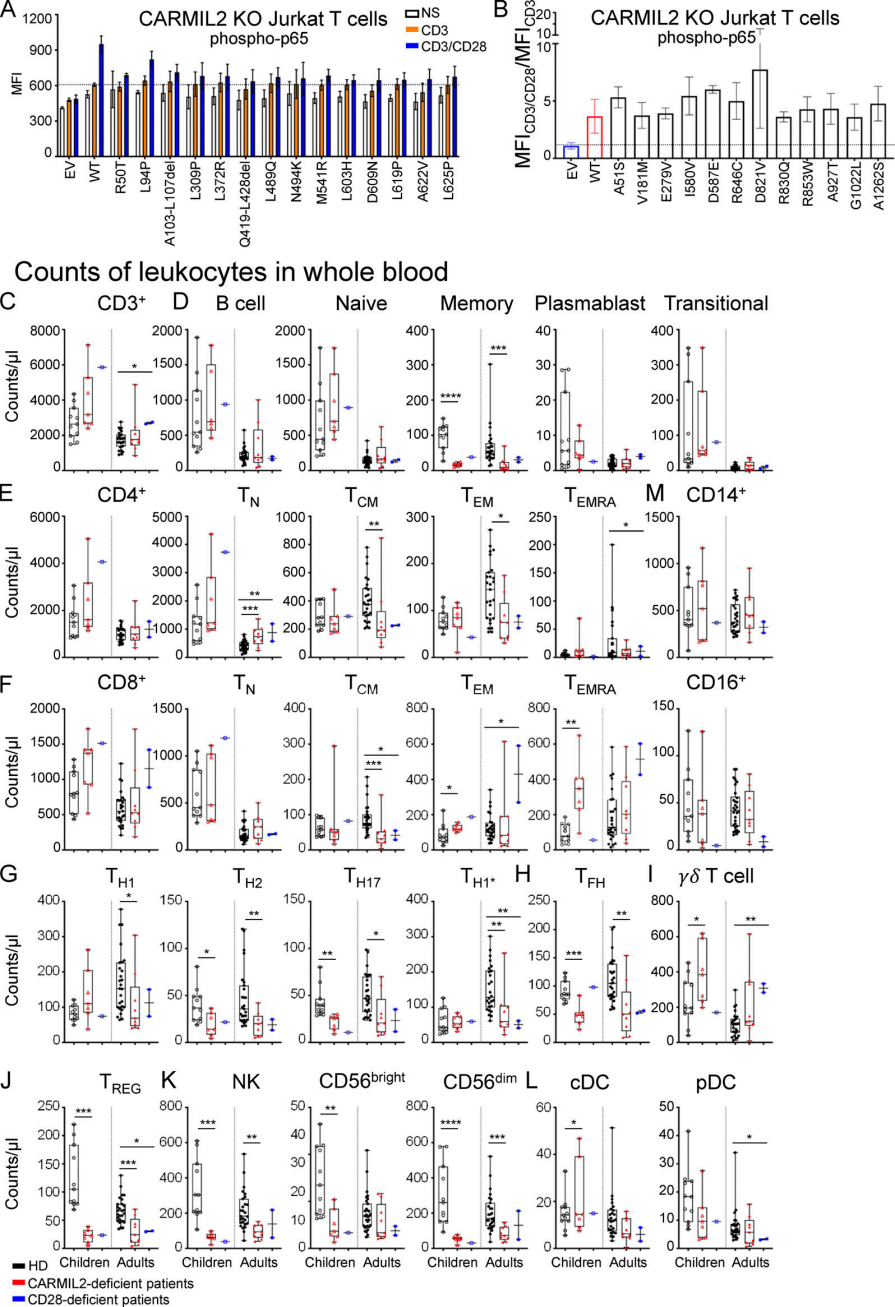
**Functional assays in Jurkat T cells, and counts of leukocytes in blood. (A)** Phospho-p65 levels in CARMIL2 KO Jurkat T cells, as assessed by intracellular FACS, after transduction with an empty lentivirus (EV), a lentivirus encoding CARMIL2 WT isoform 3 (WT), or the indicated missense mutants identified in CARMIL2-deficient individuals, in the absence of stimulation or following stimulation with the mAb indicated. MFI and histograms are shown. The bar represents the mean. Error bars represent the SD. *N* = 3. **(B)** Phospho-p65 levels in CARMIL2 KO Jurkat T cells, as assessed by intracellular FACS, after transduction with an empty lentivirus, a lentivirus encoding WT isoform 3 (WT), or homozygous missense variants found in gnomAD (MAF higher than 10^−5^, and a CADD score above 20), following stimulation with anti-CD3 mAb with or without anti-CD28 mAb. Histograms are plotted, with the bar representing the mean. Error bars represent the SD. *N* = 3. CyTOF was conducted after the exclusion of dead cells from fresh blood isolated from 38 adult and 11 pediatric healthy controls, 9 adults and 7 children with CARMIL2 deficiency, and 3 CD28-deficient patients. **(C−M)** Box-and-whisker plots are shown for the counts of each subset of leukocytes. The whiskers indicate the maximum and minimum values. The bars represent the mean value. P values indicate significant differences (in Mann–Whitney tests, or *t* tests): *, P < 0.05; **, P < 0.01; ***, P < 0.001; ****, P < 0.0001. T_N_, naive T cells; T_FH_, follicular helper T cells.

In addition to the c.1149 + 5G>C variant, two variants (c.611 + 5G>A, c.1812-7G>A) in intronic regions were predicted to alter *CARMIL2* mRNA splicing. The c.611 + 5G>A variant led to a 2 base-pair insertion, followed by a premature stop codon (p.Asp205Trpfs*4; Fig. S1 B). For the c.1812-7G>A variant, we confirmed aberrant splicing by RT-PCR followed by TA cloning of the mRNA extracted from P31 homozygous T cell blasts (Fig. S1 C). One missense (c.871G>C, p.Gly291Arg) and two synonymous (c.1128C>T and c.1578C>T) variants were also predicted to affect mRNA splicing. The c.871G>C nucleotide substitution was predicted to disrupt the essential splicing donor site of exon 11, as confirmed by exon trapping (Fig. S1 D). The synonymous variants were predicted to introduce a novel splice donor site and an ensuing frameshift deletion. By exon trapping (Fig. S1 E) or targeted RT-PCR on mRNA extracted from T cell blasts and TA cloning (Fig. S1 F), we confirmed that both variants induced aberrant *CARMIL2* splicing. *CARMIL2* mRNA levels were also very low in T cell blasts from individuals with the c.1812-7G>A (P31), and c.1578C>T (P1) alleles, consistent with nonsense-mediated mRNA decay (Fig. S1 G). We then confirmed the pathogenicity of all 12 missense variants, 2 in-frame deletions, 1 stop codon (p.Gln792*), and 2 frameshift (p.Arg520Thrfs*38 and p.His612Thrfs*20) variants. We first overexpressed the cDNA in HEK293T cells. All missense and in-frame deletion variants were normally expressed, as shown by Western blotting (Fig. 3 A). The stop and frameshift mutations were not detected with the C-terminal mAb, but a band corresponding to a truncated protein was detected with an N-terminal polyclonal antibody (Fig S1 H). We then transduced CARMIL2-deficient Jurkat T cells with the cDNA of the 12 missense, 2 in-frame deletion variants, and the WT cDNA. All the missense alleles were expressed (Fig. S1 I), albeit less strongly than the WT (25–77% of WT values), as shown by FACS (Fig. 3 B). The WT isoform 3 restored NF-κB p65 phosphorylation upon CD28 costimulation (Fig. 3 C). However, CD28 costimulation was impaired for all 14 missense and in-frame deletion variants, with a costimulatory capacity of 0–39% relative to cells complemented with the WT isoform 3 (Fig. 3 C and Fig. S2 A). By contrast, none of the reported homozygous missense variants with a MAF higher than 10^−5^ and a CADD score above 20 (Fig. S1 A) impaired CD28 costimulation (Fig. S2 B). Finally, we assessed CARMIL2 protein levels in available PBMCs or T cell blasts by FACS and/or Western blotting for the 41 homozygous or compound heterozygous variant alleles, including 9/12 missense, 4/5 nonsense, 11/13 frameshift, 2/2 in-frame deletions, 13/14 splice, and 2/2 synonymous variants (Fig. 3 D and Table S2). All the cells tested contained very little CARMIL2 protein, if any. Thus, all the variants strongly impaired *CARMIL2* mRNA synthesis, protein production, or CD28 signaling functions in vitro or ex vivo, confirming CARMIL2 deficiency in all 89 individuals.

### CARMIL2 deficiency has a broader impact on lymphocyte development than CD28 deficiency

**Figure 4.**
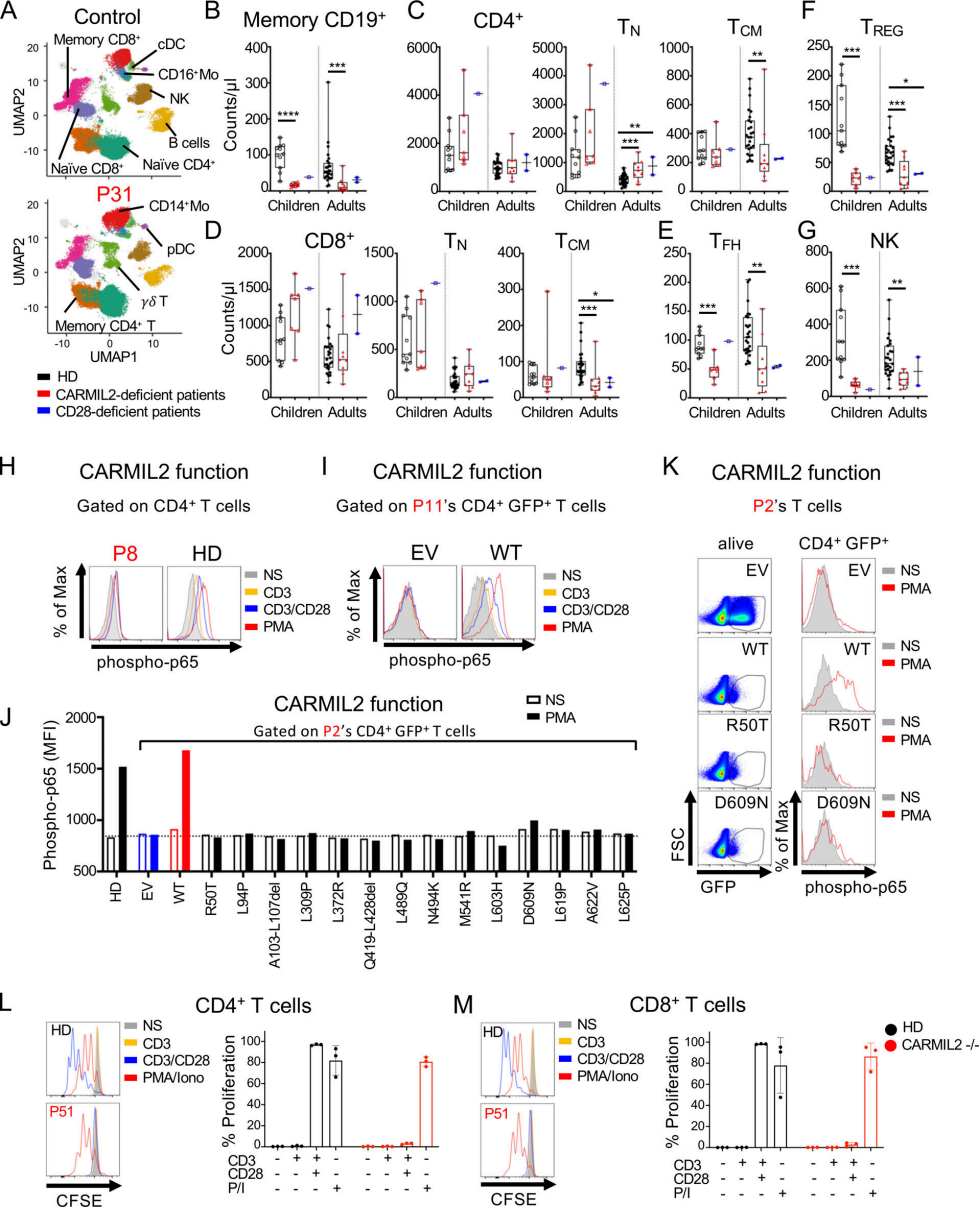
**CARMIL2 deficiency affects lymphocyte development more profoundly than CD28 deficiency.** CyTOF was conducted after the exclusion of dead cells from fresh blood isolated from 38 adult and 11 pediatric healthy controls, 9 adults and 7 children with CARMIL2 deficiency, and 3 CD28-deficient patients. **(A)** UMAP plots are presented with legends indicating the different leukocyte subsets, as defined by surface markers. **(B−G)** Box-and-whisker plots are shown for the counts of each subset of leukocytes. The whiskers indicate the maximum and minimum values, with the bars indicating the mean. P values indicate significant differences (Mann–Whitney test, or *t* test): *, P < 0.05; **, P < 0.01; ****, P < 0.0001. T_N_, naive T cell; T_FH_, follicular helper T cell. **(H)** Phospho-p65 levels, as assessed by intracellular FACS on CD4^+^ T cells from a HD and P8 (N41Kfs*47), upon stimulation with anti-CD3 mAb with or without anti-CD28 mAb, or PMA. **(I)** Phospho-p65 levels, as assessed by intracellular FACS on T cell blasts from P11 (Q817*) transduced with an empty lentivirus or a lentivirus encoding the WT isoform 3 (WT), following stimulation with anti-CD3 mAb with or without anti-CD28 mAb, or PMA. Results are shown for CD4-expressing cells also positive for GFP. **(J and K)** Phospho-p65 levels, as assessed by intracellular FACS after PMA stimulation in T cell blasts from a HD and P2 (L636Afs*39) transduced with an empty lentivirus (EV), a lentivirus encoding WT isoform 3 (WT), or the variants indicated, identified in CARMIL2-deficient individuals. Results are shown for CD4-expressing cells also positive for GFP. Representative results from two experiments are shown. **(L and M)** Proliferation of sorted naive (defined as CD3^+^ CD45RA^+^ CCR7^+^ cells) CD4^+^ (L) and CD8^+^ (M) T cells following 4 d of incubation with anti-CD3 mAb, anti-CD3 + anti-CD28 mAb, or P/I. Proliferation was assessed by CFSE dilution. Representative flow plots for P51 and a HD are depicted. The bar represents the mean. Error bars represent the SD.

**Figure S3.**
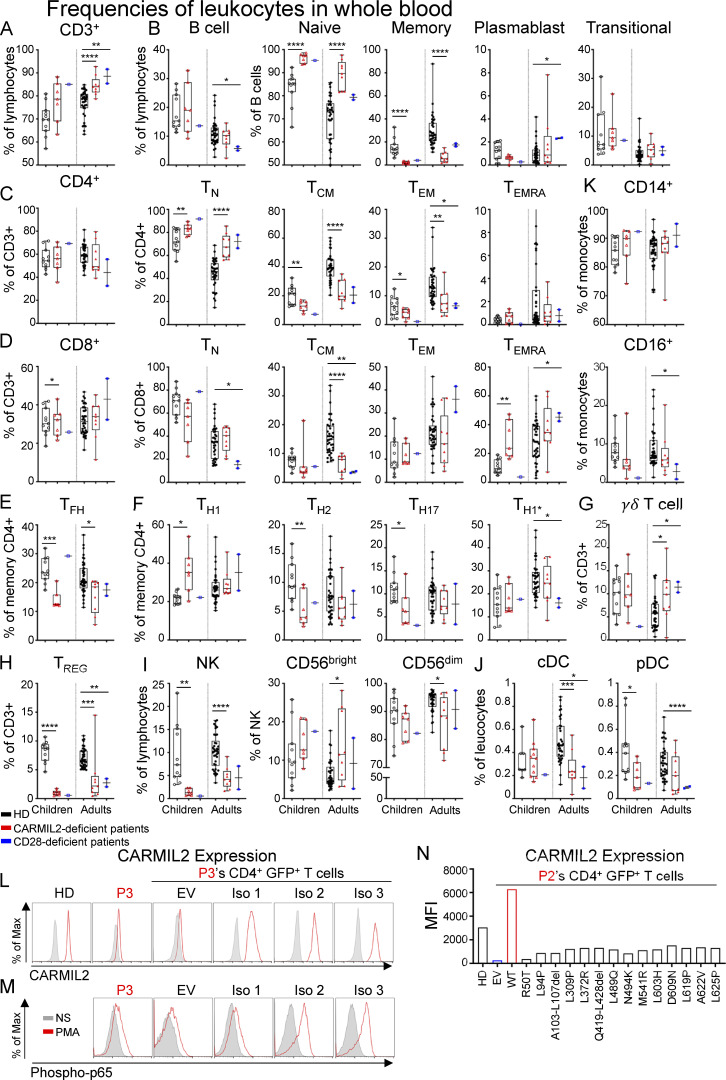
**Frequencies of leukocytes in blood, and functional assays in primary CD4^+^ T cells.** CyTOF was conducted after the exclusion of dead cells from fresh blood isolated from 38 adult and 11 pediatric healthy controls, 9 adults and 7 children with CARMIL2 deficiency, and 3 CD28-deficient patients. **(A−K)** Box-and-whisker plots are shown for the frequencies of each subset of leukocytes. The whiskers indicate the maximum and minimum values. The bars represent the mean. P values indicate significant differences (Mann-Whitney tests, or *t* tests): *, P < 0.05; **, P < 0.01; ***, P < 0.001; ****, P < 0.0001. T_N_, naive T cells; T_FH_, follicular helper T cells. **(L)** CARMIL2 levels (upper panel), as assessed by intracellular FACS on T cell blasts from a HD and P3 (L636Afs*39) transduced with an empty lentivirus (EV), or lentiviruses encoding CARMIL2 isoforms 1, 2, and 3. **(M)** Phospho-p65 levels (lower panel), as assessed by intracellular FACS on T cell blasts from P3 (L636Afs*39) transduced with an empty lentivirus (EV), or lentiviruses encoding CARMIL2 isoforms 1, 2, and 3 with or without stimulation with PMA (representative results from two experiments are shown). **(N)** CARMIL2 levels, as assessed by intracellular FACS on T cell blasts from a HD and from P2 (L636Afs*39) transduced with an empty lentivirus (EV), or lentiviruses encoding the missense and in-frame deletion variants found in CARMIL2-deficient individuals. *N* = 1.

We performed mass cytometry (CyTOF) on whole-blood samples from 38 adult (range: 22–57 yr, median age: 32 yr) and 11 pediatric (range: 1–14 yr, median age: 7 yr) HDs, 9 adult (range: 20–43 yr, median age: 25 yr), and 7 pediatric (range: 5–16 yr, median age: 10 yr) CARMIL2-deficient individuals, and 3 CD28-deficient individuals (12, 30, and 40 yr of age) to evaluate the distribution of leukocyte subsets (Fig. 4, A–G; Fig. S2, C–M; and Fig. S3, A–K). CARMIL2- and CD28-deficient individuals had normal counts of CD3^+^ T cells and CD19^+^ B cells (Fig. S2, C and D). CARMIL2-deficient adults and children had normal counts of CD19^+^CD27^−^ naive B cells, transitional B cells, and CD24^−^CD38^++^ plasmablasts (Fig. S2 D), but markedly low levels of CD19^+^CD27^+^ memory B cells (Fig. 4 B). For T cells, total CD4^+^ and CD8^+^ T cell counts were similar in all groups (Fig. 4, C and D). However, CARMIL2- and CD28-deficient adults had higher counts of naive CD4^+^ cells, whereas their central memory (T_CM_) CD4^+^ and CD8^+^ and effector memory (T_EM_) CD4^+^ subsets were smaller, with normal counts of terminally differentiated effector memory cells (T_EMRA_; Fig. 4, C and D; and Fig. S2, E and F). Within the memory CD4^+^ T cell compartment of CARMIL2- and CD28-deficient individuals, follicular helper T cell (Fig. 4 E), T_H1_, T_H2_, T_H1*_, and T_H17_ cell counts were low (Fig. S2, G and H), probably reflecting lower overall counts of memory CD4^+^ T cells. For other T cell subsets, γδ T cell counts were high in children and normal in adults with CARMIL2 deficiency (Fig. S2 I), and the counts of T_REG_, defined as CD4^+^CD25^+^CD127^low^ cells, were very low in adults and children with CARMIL2 deficiency and in CD28-deficient individuals (Fig. 4 F and Fig. S2 J). NK cell counts were low in CARMIL2-deficient adults and children (Fig. 4 G). The counts of CD56^bright^ NK cells were low in children and normal in adults, whereas the counts of CD56^dim^ NK cells were low in both adults and children with CARMIL2 deficiency (Fig. S2 K). Finally, CARMIL2- and CD28-deficient individuals had normal counts of CD14^+^ and CD16^+^ monocytes, cDCs, and pDCs (Fig. S2, L and M). Overall, these findings suggest that impaired CD28 signaling is responsible for the impaired generation and/or survival of memory CD4^+^ and CD8^+^ T cells and T_REG_s in CARMIL2-deficient individuals. By contrast, the normal generation and/or maintenance of memory B cells and NK cells in CD28-deficient individuals suggest that these specific defects in CARMIL2-deficient individuals are not related to the CD28 signaling defect.

### T cell function is impaired in CARMIL2 deficiency

**Figure S4.**
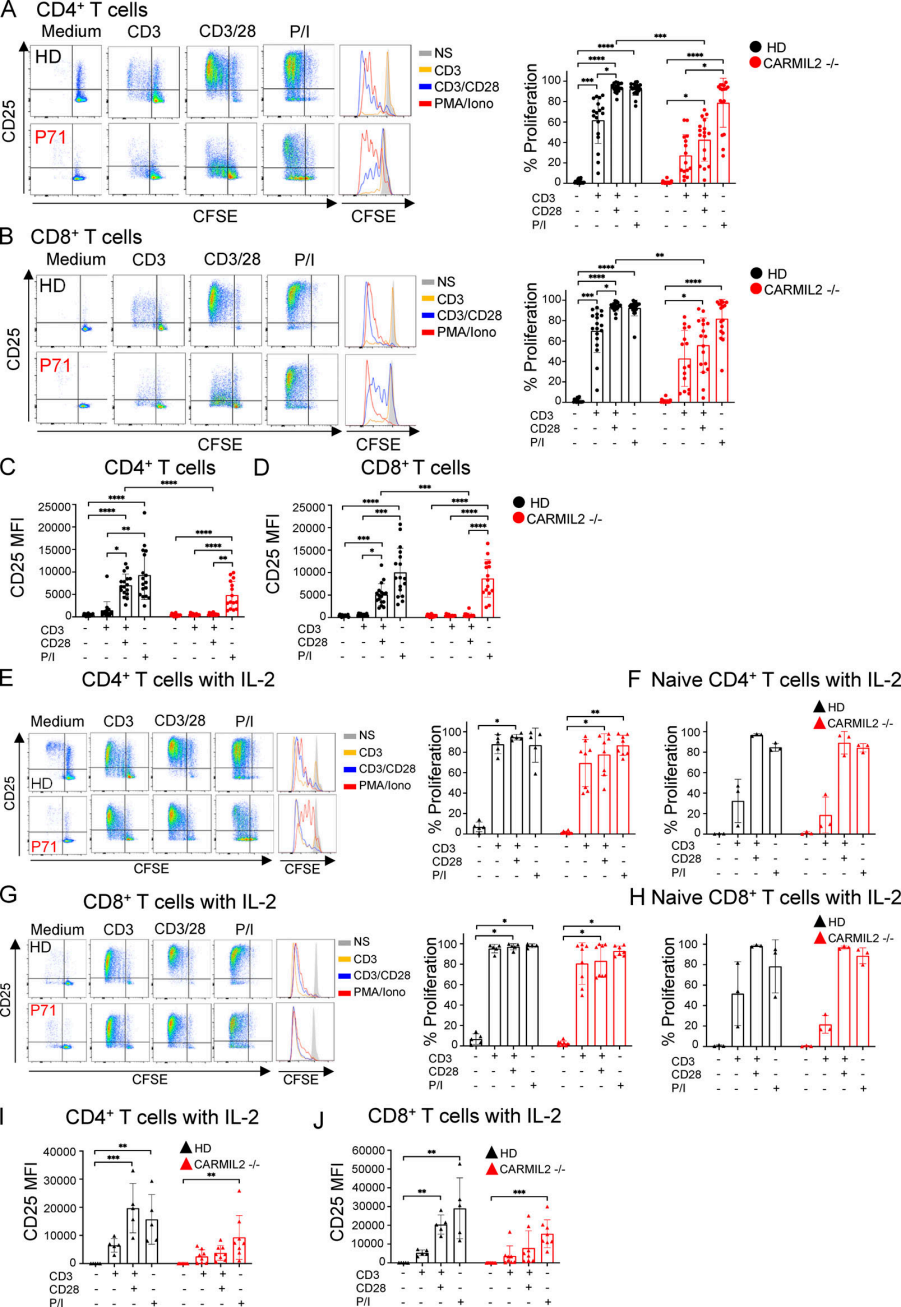
**T cell function is impaired in patients. (A and B)** T cell proliferation in CD4^+^ (A) and CD8^+^ (B) T cells from 17 CARMIL2-deficient patients and 19 HD, upon stimulation with anti-CD3 mAb, anti-CD3 + anti-CD28 mAb, or P/I. Representative flow plots for the proliferation of CD4^+^ (A) and CD8^+^ (B) T cells from P71 and a HD are shown. Proliferation was assessed by CFSE dilution. The bars represent the mean. The error bars represent the SD. P values were calculated using Kruskal–Wallis test alongside Dunn’s correction for multiple comparisons with *, P < 0.05; **, P < 0.01; ***, P <0.001; and ****, P < 0.0001. **(C and D)** MFI of CD25 in CD4^+^ T cells (C) and CD8^+^ T cells (D) from PBMCs upon stimulation with anti-CD3 mAb, anti-CD3 + anti-CD28 mAb, or P/I. **(E–H)** Proliferation of CD4^+^ T cells (E) and CD8^+^ T cells (G) within PBMCs, or sorted naive (defined as CD3^+^ CD45RA^+^ CCR7^+^ cells) CD4^+^ T cells (F) and CD8^+^ T cells (H) following 4 d of incubation with the abovementioned stimuli and the addition of IL-2 (500 IU/ml). Representative flow plots for P71 and a HD are depicted. The bars represent mean. The error bars represent the SD. **(I and J)** MFI of CD25 in CD4^+^ T cells (I) and CD8^+^ T cells (J) stimulated with additional IL-2 (500 IU/ml) in the PBMCs of eight CARMIL2-deficient patients and five HDs. The bars represent mean. The error bars represent the SD. Statistical analysis was performed with Kruskal–Wallis tests and Dunn’s correction for multiple comparisons with *, P < 0.05; **, P < 0.01; ***, P < 0.001; and ****, P < 0.0001.

We previously showed that CARMIL2 deficiency affects NF-κB activation in CD4^+^ and CD8^+^ T cells upon CD28 cosignaling (Wang et al., 2016; Schober et al., 2017). Extending this finding, we found that the PMA-driven activation of the NF-κB pathway was also impaired in CARMIL2-deficient CD4^+^ and CD8^+^ T cells (Fig. 4 H and Table S2), suggesting that PKC-θ function is dependent on CARMIL2, but in a CD28-independent manner. Although we showed that WT *CARMIL2* isoform 3 rescued NF-κB activation upon CD28 signaling in CARMIL2-deficient Jurkat T cells (Fig. 3 C), we further tested the function of documented *CARMIL2* alleles in primary T cells, as the lack of phosphatase and tensin homolog in Jurkat cells might have affected the CD28 signaling pathway due to impaired negative regulation of PI-3K signaling (Shan et al., 2000). Impaired NF-κB activation upon stimulation with PMA and CD3/CD28 was rescued in primary T cells by the transduction of CARMIL2-deficient cells with the WT *CARMIL2* isoform 3, but not by transduction with an empty vector (Fig. 4 I). Furthermore, *CARMIL2* cDNA encoding WT isoform 1 or the variants identified in the patients were expressed at a lower level and were unable to restore NF-κB activation upon PMA activation following the transduction of CARMIL2-deficient primary T cells (Fig. 4, J and K; and Fig. S3, L−N). We also tested 17 CARMIL2-deficient individuals and measured ex vivo T cell proliferation and CD25 upregulation upon stimulation with anti-CD3 mAb, anti-CD3/CD28 mAb, or PMA/ionomycin (P/I) in PBMC cultures. We observed markedly decreased CD28 cosignaling in terms of CD4^+^ and CD8^+^ T cell proliferation and CD25 upregulation, whereas the response to CD3 alone or P/I was only slightly weaker than in healthy controls (Fig. S4, A−D). To rule any contribution of APC present within the assayed PBMCs population, we sorted naive T cells and confirmed defective CD28 cosignaling in CARMIL2-deficient T cells (Fig. 4, L and M). CD28 cosignaling is required for potent and optimal IL-2 expression. We therefore investigated whether exogenous IL-2 could rescue T cell proliferation in individuals with CARMIL2 deficiency. The addition of IL-2 to PBMCs cultures incubated with anti-CD3/CD28 mAb resulted in levels of CD4^+^ and CD8^+^ T cell proliferation similar to those observed in HD cell cultures or sorted naive T cells (Fig. S4, E−H). CD25 upregulation following anti-CD3/CD28 stimulation of PBMCs was also increased by exogenous IL-2, however it did not reach the levels of healthy control cells (Fig. S4, I and J). These results confirm that CARMIL2 deficiency results in markedly impaired CD28 cosignaling, leading to lower T cell proliferation capacities, as observed in individuals with CD28 deficiency (Béziat et al., 2021), and that these phenotypes can be rescued by the addition of IL-2, at least in vitro. We also show that NF-κB activation upon CD28 crosslinking in CARMIL2-deficient T cells can be rescued by transduction with the *CARMIL2* WT isoform 3, and that PKC-θ is dependent on CARMIL2 for signaling in a CD28-independent manner.

### CARMIL2 controls the activation of NF-κB target genes activation upon PMA stimulation

**Figure 5.**
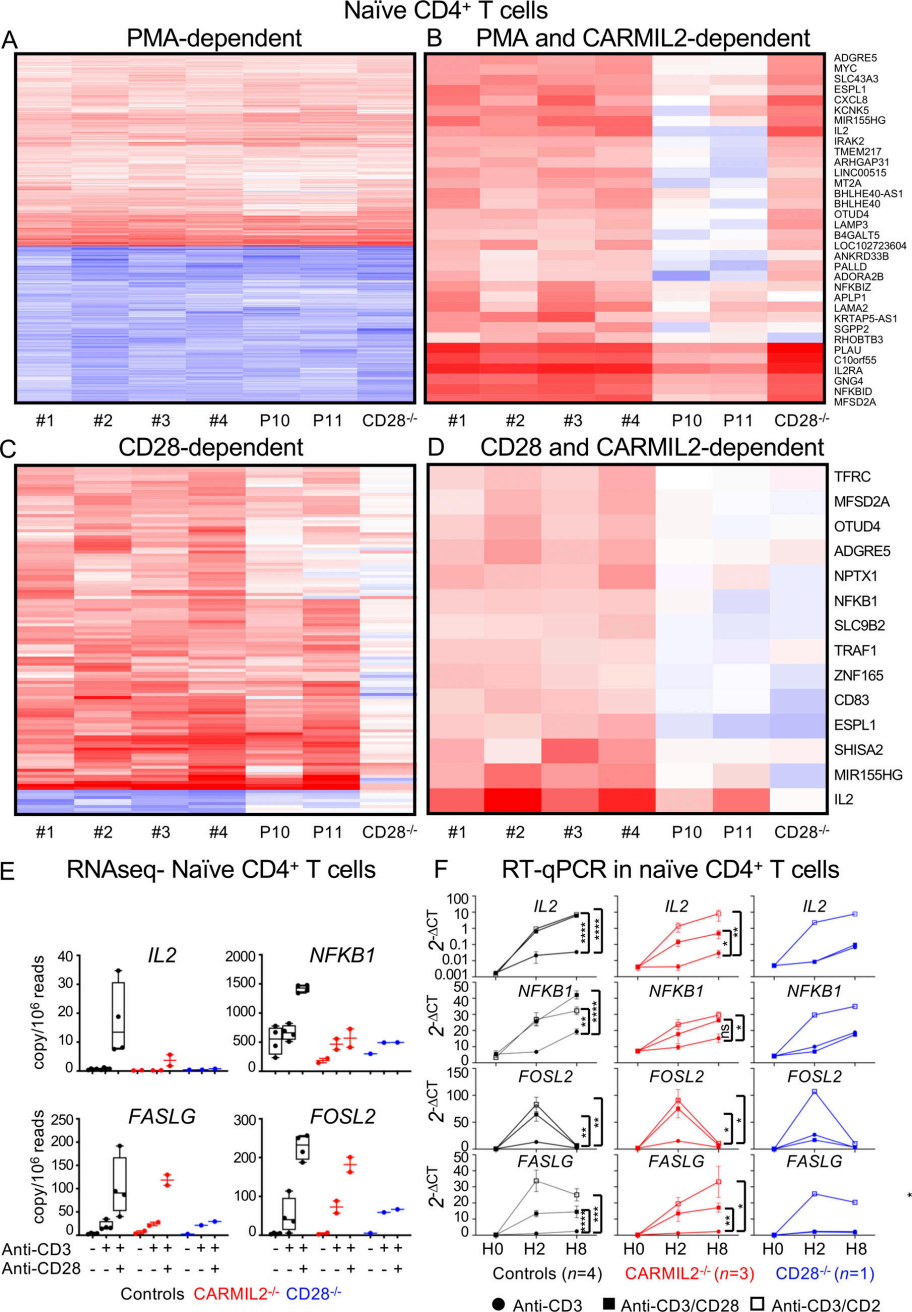
**CARMIL2 regulates the expression of a restricted set of genes downstream from CD28 signaling.** Impact of CARMIL2 deficiency on the transcriptome of sorted naive CD4^+^ T cells. RNA-seq data. Heatmap showing log_2_ FC in expression in stimulated naive CD4^+^ T cells at 2 h. **(A)** Only genes differentially expressed in response to stimulation in controls (adjusted P < 0.05 and | log_2_ FC | > 2) upon simulation with PMA are shown (PMA dependent). **(B)** Only genes both PMA dependent and differentially expressed in CARMIL2-deficient patients (adjusted P < 0.05 and log_2_ FC < −2) are shown (PMA and CARMIL2 dependent). **(C)** Heatmap showing the genes differentially expressed in control naive CD4^+^ T cells (adjusted P < 0.05 and | log_2_ FC | > 1) upon stimulation with anti-CD3 + anti-CD28 Abs versus anti-CD3 mAb alone (CD28-dependent genes). **(D)** Heatmap showing the CD28-dependent genes (defined in C) differentially expressed in CARMIL2-deficient patients relative to controls (adjusted P < 0.05 and log_2_ FC < −1). **(E)** Extraction from the RNA-seq data of representative target transcripts of the NF-κB (*NFKB1*), AP-1 (*FOSL2*), and NFAT (*FASLG*) pathways upon stimulation as indicated. **(F)** RT-qPCR on naive CD4^+^ T cells from four controls, three CARMIL2-deficient patients, and one CD28-deficient patient at 0, 2, and 8 h of activation with anti-CD3 mAb with or without anti-CD28, or with anti-CD2 mAb. Data are displayed as 2^−ΔCt^ after normalization against *GUS* expression. Statistical analysis was performed at 8 h for *IL2*, *NFKB1*, and *FASLG*, and at 2 h for *FOSL2*. Error bars represent the SEM. *, P < 0.05; **, P < 0.01; ***, P < 0.001. *N* = 1.

We characterized human CARMIL2 function at early stages of T cell activation by performing RNA-seq on purified naive CD4^+^ T cells from four controls, two CARMIL2-deficient patients, and one CD28-deficient patient 2 h after activation with PMA (Table S3; Gene Expression Omnibus [GEO] accession no. GSE169506). In naive CD4^+^ T cells from controls, 481 transcripts were upregulated and 396 transcripts were downregulated upon PMA stimulation, with a log_2_-fold change (FC) threshold of at least 2 (Fig. 5 A and Table S3; GEO accession no. GSE169506). The upregulated transcripts included those encoding the high-affinity IL-2Rα chain (ranked 10), IL-2 itself (ranked 36), NF-κB proteins, and NF-κB negative regulators (*REL* ranked 46; *NFKB1* ranked 128; *NFKBIZ* ranked 146; and *TNFAIP3* ranked 284). We identified the members of this list of top-ranking transcripts for which induction differed significantly between CARMIL2-deficient patients and controls (Fig. 5 B). The overall response to PMA did not differ between control and CD28-deficient cells, whereas CARMIL2-deficient cells had a weaker response to PMA (Fig. 5 B), with significant downregulation, by at least fourfold (log_2_FC < −2) for 35 transcripts. Many of the corresponding genes are known targets of canonical NF-κB signaling pathways, including *IL2* (ranked 1; log_2_FC: −7.3; P value: 1.1E-4), *IL2RA* (ranked 17; log_2_FC: −2.8; P value: 2.1E-38), NF-κB negative regulators, such as *NFKBID* (log_2_FC: −2.8; P value: 9.8E-31) and *NFKBIZ* (log_2_FC: −2; P value: 1.0E-6), or the proto-oncogene *MYC* (log_2_FC: −2.7; P value: 4.1E-27; Fig. 5 B). These data show that the transcriptional control of only a restricted set of target genes in response to PMA stimulation is impaired in CARMIL2-deficient T cells, including NF-κB target genes in particular.

### CARMIL2 deficiency does not impair AP1 and NFAT signaling downstream CD28

In the same RNA-seq experiment, we assessed the impact of CARMIL2 deficiency on CD3 and CD28 signaling in naive CD4^+^ T cells. We found that CARMIL2 deficiency did not impair the upregulation of 357 transcripts (log_2_FC > 1) upon CD3 stimulation alone (Table S3; GEO accession no. GSE169506) relative to controls. We then studied the CD28-dependent genes by comparing cells stimulated with anti-CD3 plus anti-CD28 mAb with cells stimulated with anti-CD3 mAb alone (Fig. 5 C). CD28-deficient cells did not respond to CD28 costimulation, but we identified 113 upregulated transcripts (log_2_FC > 1; P value: <0.05) following CD28 costimulation in control cells (Fig. 5 C), with *IL2* the top-ranked CD28-dependent transcript (log_2_FC: 5.6). Next, among the top-ranking transcripts in the controls, we identified those for which induction differed significantly between CARMIL2-deficient and control cells (log_2_FC < −1; P value: <0.05). 14 transcripts had a lower levels of expression in the patients’ cells, and many of these transcripts corresponded to known target genes of canonical NF-κB signaling pathways, including *IL2* (log_2_FC: −2.2; P value: 6.5E-3), *NFKB1* (log_2_FC: −1.2; P value: 2.1E-6), *CD83* (log_2_FC: −1.3; P value: 3.2E-6), and *TRAF1* (log_2_FC: −1.2; P value: 4.2E-8; Fig. 5 D). CD28 is known to activate three main pathways: the NF-κB, AP-1, and NFAT pathways (Esensten et al., 2016). Upon CD28 costimulation, CARMIL2-deficient cells displayed normal upregulation of *FASLG* and *FOSL2*, two prototypic target genes of NFAT and AP-1, respectively (Fig. 5 E). A partial impairment of CD28 signaling in CARMIL2-deficient cells with intermediate levels of *IL2* and *NFKB1* induction and preserved induction of *FOSL2* and *FASLG* were confirmed by RT-qPCR in isolated naive CD4^+^ T cells from three CARMIL2-deficient patients (Fig. 5 F). In addition, *IL2* expression by CARMIL2-deficient cells was rescued by CD2 costimulation, as shown by RT-qPCR (Fig. 5 F). Thus, CARMIL2 deficiency only partially impairs CD28 signaling by affecting the NF-κB pathway, but sparing the AP-1 and NFAT pathways.

### B cell function is impaired in individuals with CARMIL2 deficiency

**Figure 6.**
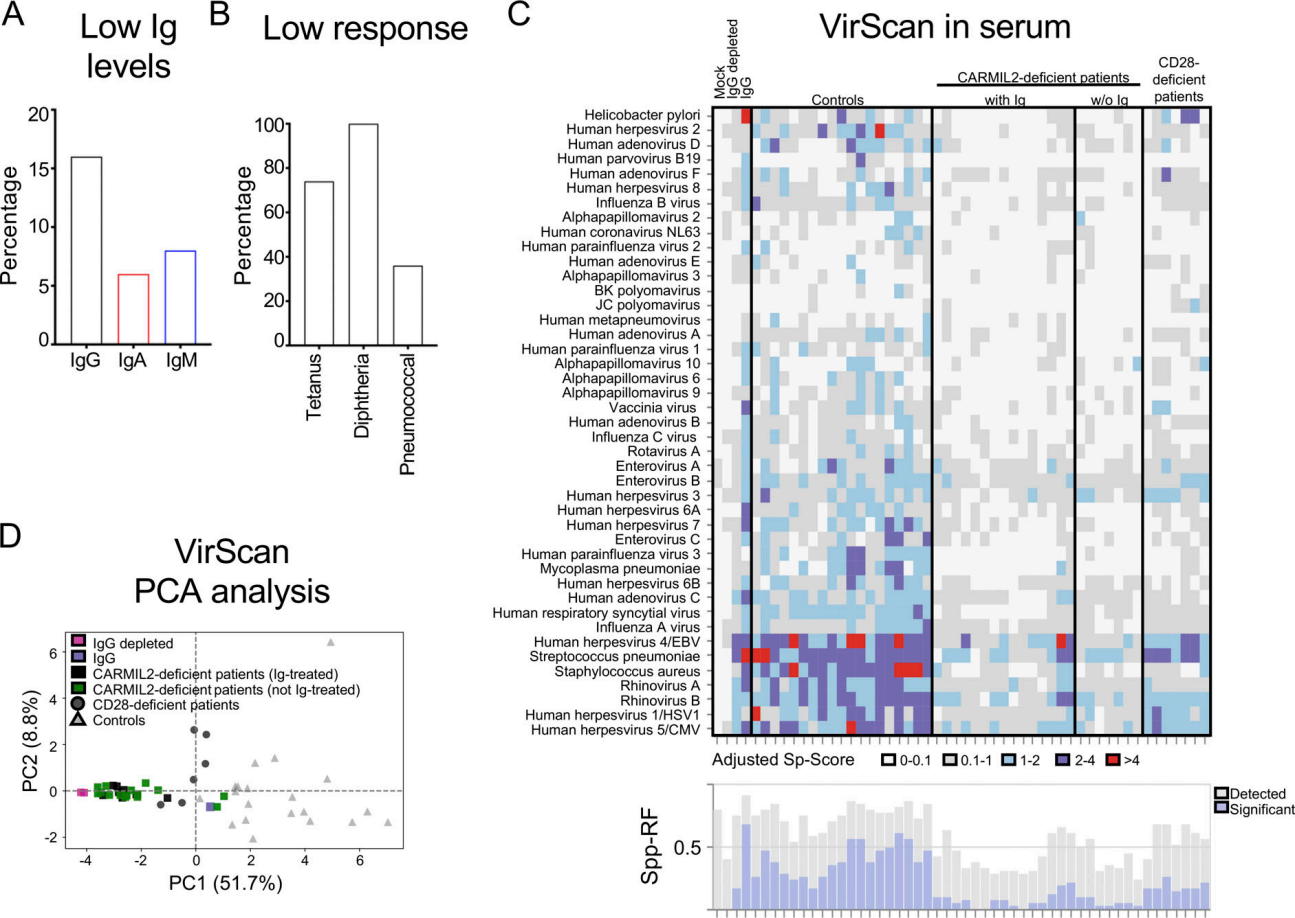
**B cell function is impaired in individuals with CARMIL2 deficiency. (A)** Frequencies of CARMIL2-deficient patients with serum Ig levels (IgG, IgA, and IgM) below the normal range. **(B)** Frequencies of CARMIL2-deficient patients with low serological titers of Abs against booster vaccines for tetanus toxoid, diphtheria, and pneumococcus. **(C)** Adjusted virus scores (Virus Scoreadj) for samples from 20 adult in-house controls, 23 CARMIL2-deficient patients, and 7 CD28-deficient patients, together with mock IP samples, IgG-depleted serum, and IVIg. Virus species for which at least one sample was seropositive (i.e., with Ab levels above our species-specific significance cutoff) are shown (y axis). The heatmap plot shows the z-score values for each sample on a color gradient; in blue if Abs were detected but the counts of non-homologous enriched peptides were below our significance cutoff values, and in purple to red if the z-score values were at least 1× higher (purple) or >2× higher (red) than our significance cutoff values. The bar plot on the bottom illustrates the size of the Ab repertoire for a given sample: the precise number of different species for which peptide enrichment was observed (gray) and the number of different species for which the z-score values passed the significance cutoff (blue). **(D)** Principal component analysis (PCA) scatter plot for virus scores, color-coded for different groups of samples.

Despite normal total B cell counts, the CARMIL2-deficient individuals had low CD19^+^CD27^+^ memory B cell levels (Fig. 4 B). We analyzed the impact of this defect on B cell function and found that serum IgG concentrations were within the normal range in most CARMIL2-deficient individuals, as only 12/80 (15%) presented hypogammaglobulinemia (Fig. 6 A and Table S4). Similarly, IgM and IgA concentrations were low in only 6/79 (8%) and 4/71 (6%) of these individuals, respectively. Despite generally normal Ig concentrations, CARMIL2-deficient individuals displayed abnormally weak-specific Ab responses to protein-based booster vaccines, as 23/32 (72%) and 15/15 (100%) of the patients had low titers or no Abs against tetanus and diphtheria toxoid, respectively (Fig. 6 B and Table S4). By contrast, a serological response to polysaccharide pneumococcal vaccine was detectable in 16/25 (64%) of the patients. We then assessed the serum concentrations of Abs against various pathogens by VirScan (Xu et al., 2015) in 23 CARMIL2-deficient individuals. Global specific Ab responses were found to be weak (Fig. 6 C). Principal component analysis confirmed a clustering close to the negative control (Fig. 6 D), suggesting that CARMIL2-deficient individuals mount and/or maintain only weak specific Ab responses to pathogens. Some CARMIL2-deficient individuals also had high serum IgE concentrations (27%; Table S4), consistent with the frequent allergic manifestations described below. In summary, although CARMIL2-deficient individuals generally have normal serum Ig concentrations, abnormal B cell function leads to impaired specific Ab responses to a broad range of pathogens.

### Initial clinical presentation of CARMIL2 deficiency

**Figure 7.**
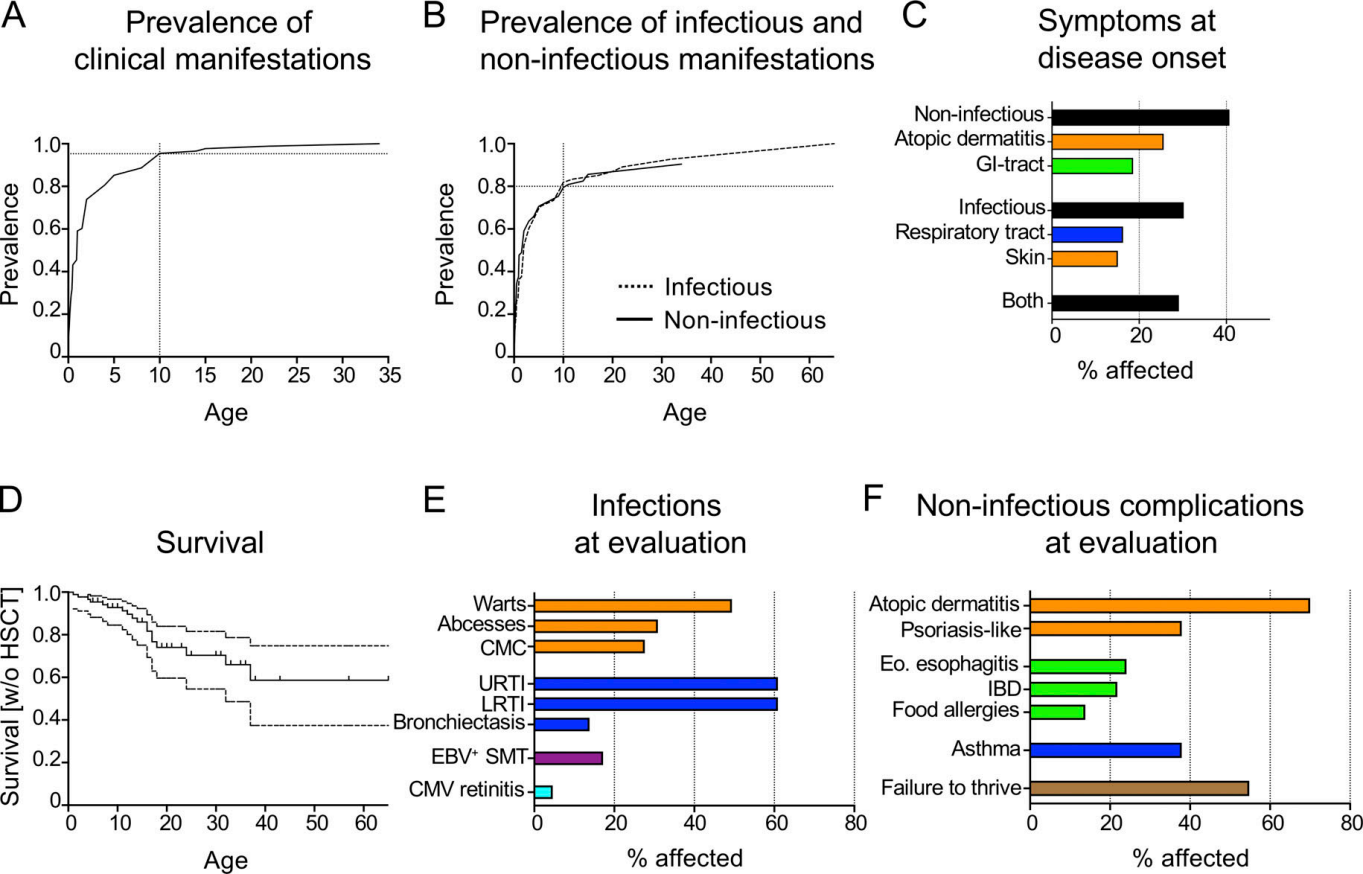
**Clinical manifestations of CARMIL2 deficiency. (A and B)** Penetrance of clinical symptoms (A) and of infectious versus non-infectious complications (B) by age in 86 CARMIL2-deficient patients. **(C)** Clinical description of the first symptoms at disease onset. **(D)** Kaplan–Meier curve depicting the survival of CARMIL2-deficient patients who did not undergo hematopoietic stem cell transplantation, with the small vertical bars indicating age at last follow-up. The dashed lines indicate 95% confidence intervals. **(E and F)** Clinical description of infectious complications (E) and non-infectious complications (F) in 87 CARMIL2-deficient individuals at evaluation. CMC, chronic mucocutaneous candidiasis; URTI, upper respiratory tract infections; LRTI, lower respiratory tract infections; Eo, eosinophilic.

A clinical description of first symptoms was available for 86/89 (97%) CARMIL2-deficient individuals. Median age at symptom onset was 1 yr (range: 0–22 yr; mean: 2.8 yr). Penetrance reached 95% at 10 yr of age (Fig. 7 A). Both infectious and non-infectious manifestations had affected 80% of patients by the age of 10 yr (Fig. 7 B). However, an assessment of the first symptoms in this cohort indicated that isolated non-infectious manifestations were common, occurring in 35 of 86 (41%) CARMIL2-deficient individuals. Atopic dermatitis (26%), starting at a median age of 0.5 yr (range: 0–34 yr), and gastrointestinal (GI) involvement (19%), presenting as chronic diarrhea at a median age of one year (range: 0–15 yr), were the most frequently reported manifestations. The earliest signs of susceptibility to infection were recurrent respiratory tract infections (16%, median age: 2 yr, range: 0–9 yr) and skin infections (15%, median age: 2 yr, range: 0–22 yr), including chronic mucocutaneous candidiasis, viral warts, and bacterial abscesses (Fig. 7 C). Our analysis of the long-term consequences of CARMIL2 deficiency revealed a progressive decline in survival that was most pronounced during adolescence (Fig. 7 D). By the age of 18 yr, 26% of CARMIL2-deficient individuals had either succumbed to disease or undergone hematopoietic stem cell transplantation. In summary, the penetrance of CARMIL2 deficiency was almost complete before the age of 10 yr, with both infectious and non-infectious manifestations. CARMIL2 deficiency was also associated with high mortality, particularly during the teenage years.

### Infectious manifestations

We retrieved detailed infectious phenotype descriptions for 87/89 (98%) CARMIL2-deficient individuals (Fig. 7 E). 84 of 87 patients (97%) had viral, bacterial, mycobacterial, or fungal infections. Mucocutaneous infections were reported in 64/87 (74%) CARMIL2-deficient individuals, with viral pathogens the most frequently detected. 43 of 87 (49%) CARMIL2-deficient individuals suffered from cutaneous flat or common warts, molluscum contagiosum, and HSV or varicella zoster virus infections or reactivations. Bacterial skin abscesses occurred in 26/87 (30%), and chronic mucocutaneous candidiasis, presenting as oral thrush, intertrigo, onychomycosis, and/or esophagitis, occurred in 24/87 (28%) CARMIL2-deficient individuals. Recurrent upper and lower respiratory tract infections were noted in 53/87 (61%) and 53/87 (61%) CARMIL2-deficient individuals, respectively (Fig. 7 E). The pathogen most frequently isolated from respiratory tract specimens was CMV, in 10/52 patients (19%; Table S5). A broad spectrum of bacterial (*Streptococcus pneumoniae*, *Haemophilus influenzae*, *Pseudomonas aeruginosa*, *Klebsiella pneumoniae*, *Staphylococcus aureus*, *Nocardia* spp., and *Neisseria flavescens*), mycobacterial (*Mycobacterium avium*, *Mycobacterium tuberculosis*), and fungal (*Aspergillus* spp.) pathogens were reported (Table S5). Bronchiectasis was reported in 12/87 (14%) CARMIL2-deficient individuals. Ongoing CMV-DNA viral replication was found in 54% of (29/54) patients tested. In addition to CMV pneumonia, five cases of CMV-induced retinitis and four cases of CMV colitis were reported, so clinical CMV disease was observed in 11/87 (13%) CARMIL2-deficient individuals. In two thirds (40/62, 65%) of tested CARMIL2-deficient individuals, chronic EBV replication was detectable in the blood. EBV^+^ SMTs were reported in 15/87 (17%) CARMIL2-deficient individuals. Other rare infections included one case of varicella zoster virus–associated cerebral vasculitis (P4), two of BK virus cystitis (P18, P20), one disseminated brain and spine *Mycobacterium chelonae* infection (P34), and one case of visceral leishmaniasis (P73). CARMIL2-deficient individuals therefore suffered from a plethora of infectious diseases.

### EBV^+^ SMTs

15 of 87 (17%) CARMIL2-deficient individuals aged 6–37 yr at evaluation had EBV^+^ SMTs documented by histopathology and EBV-encoded small RNAs in situ hybridization (Magg et al., 2018; Table S6). Pathological findings typically included a spindle-shaped cell morphology with eosinophilic cytoplasm and elongated nucleoli, the expression of smooth muscle differentiation markers and positivity for EBV-encoded small RNAs. EBV^+^ SMTs occurred at various anatomical sites, mostly within the GI tract and the liver (Table S6). They were also detected in the adrenal glands, lungs, and, less frequently, spleen, kidneys, pancreas, brain, and bones (Table S6). EBV viremia was not detected in 3/15 affected individuals (20%) and an absence of viremia should not, therefore, be regarded as an exclusion criterion for EBV^+^ SMTs (Magg et al., 2018). Serological testing revealed anti-VCA IgG in all nine patients tested, and three of these individuals also had anti-VCA-IgM. Incomplete seroconversion was also evident, as anti-EBNA Abs were detected in only one of eight individuals. It therefore appears important to screen CARMIL2-deficient individuals for the presence of EBV^+^ SMTs by whole-body imaging techniques, ideally full-body magnetic resonance imaging, because blood tests are unable to detect these tumors. During the follow-up period, progressive EBV^+^ SMTs accounted for four deaths. Consistent with published findings, no other malignancies were identified in this cohort.

### Inflammatory manifestations

Atopic dermatitis affected 60/87 (69%) CARMIL2-deficient individuals within the first 2 yr of life (Fig. 7 F). In addition, psoriasis-like lesions were noted in 33/87 (38%) individuals. Other rare skin manifestations included vitiligo and pyoderma gangrenosum, in one patient each (P77 and P53, respectively). GI manifestations were reported in 55/87 (63%) CARMIL2-deficient individuals, of whom 19/87 (22%) had histologically confirmed inflammatory bowel disease (IBD). Most IBD manifestations occurred before the age of 6 yr and were therefore classified as very early-onset IBD (Ouahed et al., 2020). Eosinophilic enteropathy, usually manifesting as esophagitis, was observed in 21/87 individuals (24%). The cohort analysis, thus, revealed a largely unrecognized phenotype of CARMIL2 deficiency. In nine individuals, upper GI tract involvement led to esophageal, pyloric, or duodenal stenosis. Failure to thrive was noted in 46/84 (55%) CARMIL2-deficient individuals and was positively associated with GI tract involvement (r = 0.31, P < 0.01). Food allergies were reported in 12/87 (14%) and allergic asthma was diagnosed in 33/87 (38%) CARMIL2-deficient individuals. Non-infectious manifestations frequently appeared early in life, predominantly affected the mucosal and cutaneous barriers, and caused relevant morbidity.

### *CARMIL2* somatic reversions are associated with a milder disease course

**Figure 8.**
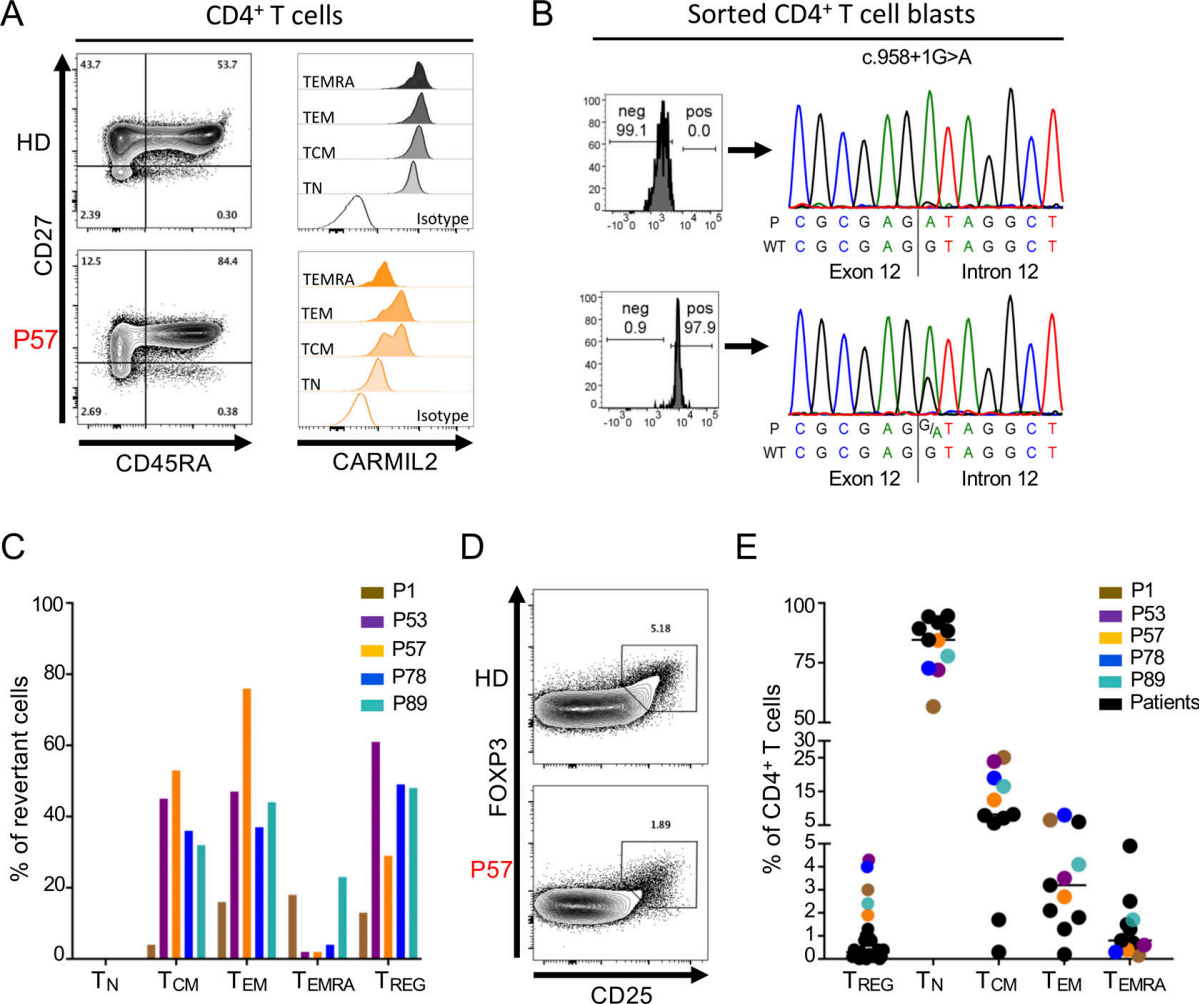
**Evidence for somatic reversions. (A)** FACS plots (left) and histograms (right) showing CARMIL2 expression in the subsets of naive and memory CD4^+^ T cells, as defined by the surface expression of CD27 and CD45RA, in P57 and a HD. **(B)** Electropherograms show the DNA sequences in the sorted CARMIL2-negative (upper panel) and CARMIL2-expressing (lower panel) T cell blasts from P57. We detected a somatic reversion at the c.958 + 1 locus in the CARMIL2-expressing cells. **(C)** Frequencies of CARMIL2-expressing revertant naive and memory CD4^+^ T cells in five patients with CARMIL2 deficiency, as defined by the surface expression of CD27 and CD45RA, determined by FACS. **(D)** FACS plots showing T_REG_, as defined by the expression of CD25 and FOXP3, in P57 and a HD. **(E)** Frequencies of T_REG_, naive (T_N_), and subsets of memory CD4^+^ T cells in CARMIL2-deficient patients with (colored dots) and without (black dots) somatic reversion events.

**Figure S5.**
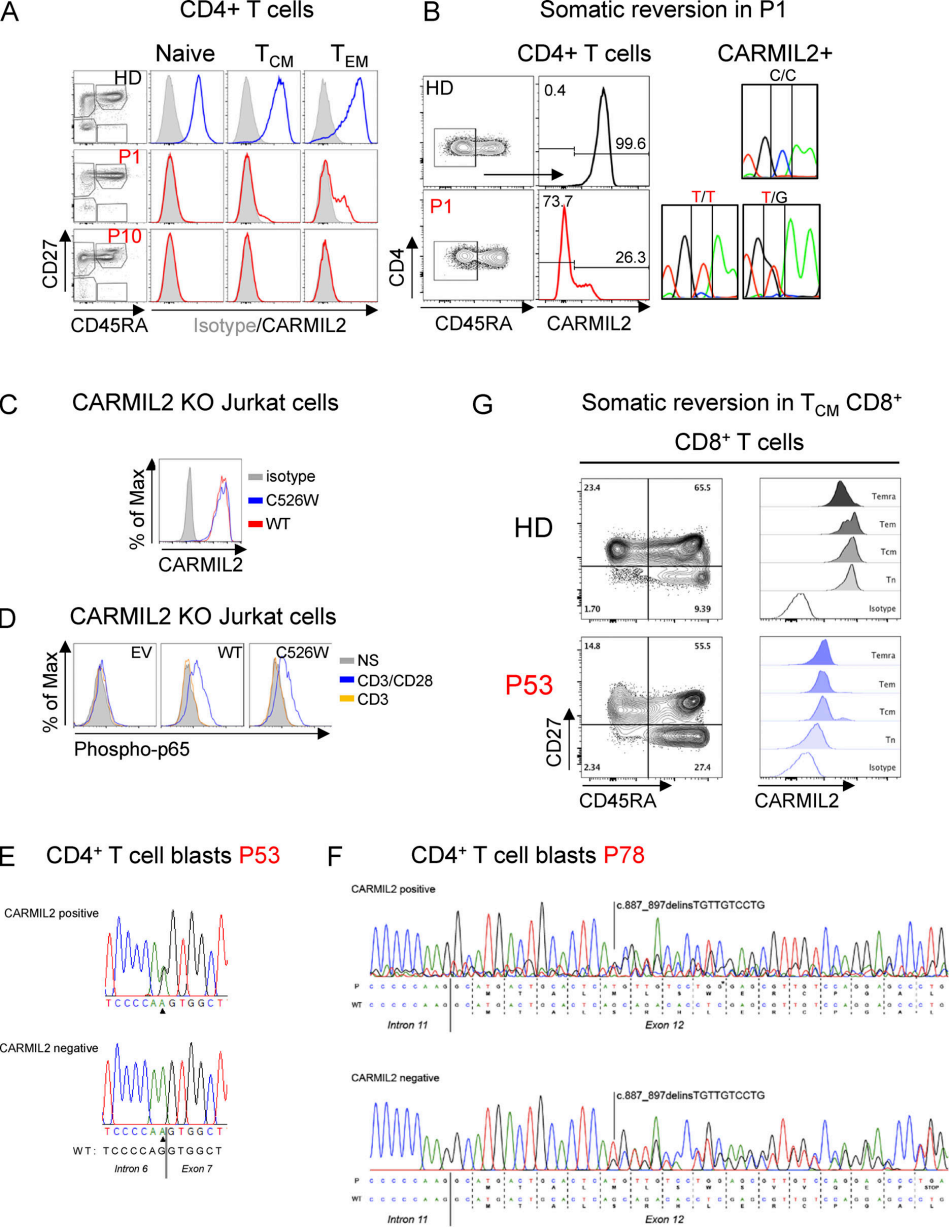
**Validation of CARMIL2 somatic reversions. (A)** CARMIL2 protein levels in PBMCs subsets of CD4^+^ T cells from a HD, P1 (c.1578C>T), and P10 (Q817*). A somatic event is suspected in CD45RA^−^CCR7^+^ T_CM_ and CD45RA^−^CCR7^−^ T_EM_ CD4^+^ T cells from P1 as shown by comparison with CD45RA^+^CCR7^+^ naive CD4^+^ T cells. **(B)** Sanger sequencing of sorted CD45RA^−^CARMIL2^+^ cells from P1 confirmed a somatic heterozygous C>G substitution at the c.1578 locus (p.C526W). **(C)** CARMIL2 protein levels in Jurkat T cells, as assessed by intracellular FACS, following transduction with an empty lentivirus (EV), WT isoform 3 (WT), or the p.C526W missense mutant. MFI is shown. **(D)** Phospho-p65 in Jurkat T cells, as described in C, following stimulation with anti-CD3 mAb with or without anti-CD28 mAb. MFI is shown. *N* = 2. **(E)** Heterozygous reversion to the WT sequence in CARMIL2-reexpressing CD4^+^ T cell blasts from P53. **(F)** Restoration of the reading frame in CARMIL2-reexpressing CD4^+^ T cell blasts from patient 78 through insertion of an additional nucleotide (G*) at cDNA-position 897, which is absent in the T cell blasts without CARMIL2 expression. **(G)** Evidence of somatic reversion in the CD8^+^ T_CM_ compartment of P53.

We found that five individuals (P1, P53, P57, P78, P89) expressed CARMIL2 in a subset of memory T cells. The levels of CARMIL2 in these cells were about 50% of those in HDs, and similar to those in healthy heterozygous family members (Fig. 3 D). This finding implies that a fraction of memory T cells had undergone somatic reversion on one allele. Two individuals displayed a heterozygous reversion to the WT allele (P53, P57), and P1 had a heterozygous somatic missense variant at the site of the mutation (c.1578C>G, p.Cys526Trp) restoring normal splicing and CD28 signaling (Fig. 8, A and B; and Fig. S5, A−E). In P78, compound heterozygous for a missense variant (p.1874T>C) and a frameshift-causing indel (c.887_897delinsTGTTGTCCTG), we observed no reversion event at the locus of the missense variant. Instead, the CARMIL2-reexpressing CD4^+^ T cells showed the insertion of an additional nucleotide at the indel-site (c.887_897delinsTGTTGTCCTGG), thereby restoring the reading frame with only amino acids 296 to 299 showing a sequence alteration (Met-Leu-Ser-Trp instead of Ser-Arg-His-Leu; Fig. S5 F). Interestingly, CARMIL2-expressing revertant T cells were found among all memory CD4^+^ T cell subsets, including T_CM_, T_EM_, and T_EMRA_ cells, at various frequencies (Fig. 8 C). We also detected near-normal frequencies of T_REG_ cells in these individuals (Fig. 8, D and E). Additionally, in P53, we detected a small proportion of CARMIL2-expressing CD8^+^ T_CM_ cells (Fig. S5 G), but no reversion events in NK or B cells. Reversion events were associated with higher CD4^+^ T_CM_ frequencies than in CARMIL2-deficient individuals without somatic reversions (Fig. 8 E). Interestingly, four of the five individuals with CD4^+^ reversion events had a mild course of disease (P53, P57, P78, and P89), and three of these patients, P53, P57, and P89, were among the oldest patients in the cohort (32, 65, and 45 yr old, respectively), suggesting that the reversion event may have been clinically beneficial (Pillay et al., 2021). Similarly, we observed a gradual improvement of disease over time in P1, following revertant inflation in CD4^+^ T_CM_ cells, increasing from 8% in 2020 to 23% at the last evaluation in 2021. Notably, P1’s severe stenosing eosinophilic esophagitis resolved, but the EBV viremia and warts remained. Thus, somatic reversion events occur in CARMIL2 deficiency, may complicate diagnosis, and may be associated with a milder disease course, presumably by rescuing the generation of memory CD4^+^ T cells and T_REG_.

## Discussion

In 2013, CARMIL2 was recognized as a cytosolic protein essential for CD28 signaling in murine T cells (Liang et al., 2013). A few years later, we reported patients with inherited CARMIL2 deficiency, documenting the essential role of CARMIL2 in the human CD28 signaling pathway (Schober et al., 2017; Wang et al., 2016). CARMIL2-deficient Jurkat T cells were complemented with a murine *Carmil2* cDNA (Roncagalli et al., 2016), but CD28 signaling was never rescued with any human *CARMIL2* cDNA. Two human isoforms are reported in Ensembl (isoforms 1 and 2). We show here that these two isoforms are either not expressed (isoform 1) or constitute a small minority form (isoform 2) in all human circulating leukocyte subsets. We show instead that a third isoform (isoform 3), absent from the Ensembl database, is expressed not only in T cells (Park et al., 2017; Uchida et al., 2021), but also in all other leukocyte subsets. This third isoform retains exon 36 and splices out an in-frame segment of exon 14. Exogenously expressed isoforms 2 and 3 are functional and can rescue CARMIL2-deficient T cells, but isoform 1 is LOF. This information is particularly important, because isoform 1 was previously used as the reference isoform for the mapping of all reported *CARMIL2* variants. Our data demonstrate that isoform 3 should be regarded as the canonical isoform and used for the mapping of *CARMIL2* variants. These results also redefine the amino-acid sequence of human CARMIL2 and, therefore, its tertiary structure. This fundamental knowledge will be crucial for future studies addressing the human CARMIL2 interactome and dissecting its functional roles in human immunity and beyond.

Our results confirm all the salient observations made in Carmil2-deficient mice. In both humans and mice, CARMIL2 is expressed in pDCs and lymphoid cells, is not essential for the development of myeloid cells, but is essential for the development of memory CD4^+^ and CD8^+^ T cells, T_REG_, T_H1_, T_H17_, and memory B cells (Liang et al., 2013; Wang et al., 2016; Schober et al., 2017; Roncagalli et al., 2016). Furthermore, in-depth studies of circulating lymphoid cells have shown that CARMIL2 is also required in humans, for the development and/or survival of follicular helper T and NK cells. The putative function of CARMIL2 in human or mouse pDCs remains unknown, but our study confirms that, as already shown in mice (Roncagalli et al., 2016; Liang et al., 2013), human CARMIL2 is required for CD28-mediated NF-κB activation in CD4^+^ and CD8^+^ T cells (Liang et al., 2013; Roncagalli et al., 2016; Wang et al., 2016; Schober et al., 2017) and for IL-2 production, probably accounting for the lower proliferation capacities of the patients’ T cells and lower frequencies of T_REG_ (Malek and Castro, 2010) and memory T cells (Farber et al., 2014). This last observation is also confirmed by our results showing that somatic reversion events rescuing CARMIL2 expression also result in higher frequencies of T_REG_ and memory T cells. By contrast to the mouse protein, human CARMIL2 is also required for NF-κB activation by surface IgM in B cells (Wang et al., 2016). However, both humans and mice lacking functional CARMIL2 have poor Ab responses to T cell–dependent antigens in vivo (Roncagalli et al., 2016; Wang et al., 2016). We also previously documented poor T cell–independent Ab responses in human patients, a finding not reported for Carmil2-deficient mice (Wang et al., 2016; Roncagalli et al., 2016). In this study of a larger cohort of patients, we report that Ab responses to polysaccharide antigens (i.e., T cell–independent antigens) are only marginally affected.

PKC-θ has been shown to associate with CD28 in an interaction mediated by lymphocyte-specific protein tyrosine kinase and the PYAP cytoplasmic motif of CD28, resulting in the recruitment of these molecules to the immunological synapse (Kong et al., 2011). PKC-θ then phosphorylates the autoinhibitory linker region of CARD11 (Matsumoto et al., 2005), leading to nucleation of the CBM-complex, resulting in coupling of the TCR and CD28 to the canonical NF-κB pathway (Gaide et al., 2002; Wang et al., 2002). The TCR and CD28 have also been shown to recruit different molecular components to activate the NF-κB pathway. The TCR uses LAT/ADAP, which associates with CARD11 (Medeiros et al., 2007), whereas CD28 requires GRB2/VAV1 to activate NF-κB signaling independently of the TCR (Thaker et al., 2015). In murine T cells, mutations affecting the LRR domain of CARMIL2 abolish the ability of CD28 to form microclusters with PKC-θ at the immunological synapse, and to recruit PKC-θ and CARD11 to the immunological synapse (Roncagalli et al., 2016). Consistent with this finding, CD28, CARD11, GRB2, VAV, and ADAP are all members of the murine thymocyte CARMIL2 interactome following stimulation with the protein tyrosine phosphatase inhibitor pervanadate (Roncagalli et al., 2016). Accordingly, both CD28- and CARMIL2-deficient human T cells display impaired canonical NF-κB signaling upon CD28-stimulation, and their IL-2 production is almost abolished (Béziat et al., 2021; Wang et al., 2016; Schober et al., 2017). By comparing transcriptomic data for CD28-dependent signals in WT, CD28-deficient, and CARMIL2-deficient primary human naive CD4^+^ T cells, we show that most of the transcripts in CARMIL2-deficient cells were regulated normally in response to CD28 costimulation, with the exception of NF-κB target genes. Similarly, the response of CARMIL2-deficient naive CD4^+^ T cells to PMA was normal, except for NF-κB target gene induction. Our observations therefore suggest that PKC-θ requires CARMIL2 to activate NF-κB signaling, but that CARMIL2 is dispensable for other types of PKC-θ–dependent signaling. Given the complex clinical phenotype of human CARMIL2 deficiency, as opposed to human CD28 deficiency, our data further suggest that other PKC-θ–dependent receptors may be affected by CARMIL2 deficiency. Additional studies are warranted for the precise dissection of the spatiotemporal dynamics and biochemical requirements of CARMIL2 scaffolding functions, interaction partners, and binding motifs.

We recently reported the first description of complete CD28 deficiency in humans (Béziat et al., 2021). Unlike CARMIL2 deficiency, CD28 loss has a modest impact on the homeostasis of leukocyte subsets, as CD28- and CARMIL2-deficient patients have only low frequencies of memory CD4^+^ and CD8^+^ T cells and T_REG_ (Béziat et al., 2021) in common. CD28 deficiency has no effect on T_H_ subset differentiation or the development of NK and memory B cells (Béziat et al., 2021). Overall, the discovery of CARMIL2 deficiency is inseparable from its related function in CD28 cosignaling, but the discovery of complete CD28 deficiency in humans and the evidence of different impacts of these deficiencies on leukocyte distribution and function argue for a pleiotropic function of CARMIL2 in human, and probably mouse immunity. Consistent with this hypothesis, the clinical descriptions of CD28 and CARMIL2 deficiencies differ considerably. Autosomal recessive CD28 deficiency results in a high susceptibility to cutaneous ⍺- and γ-HPVs, including a case of “tree man” syndrome (Béziat et al., 2021). Although the discovery of other patients with CD28 deficiency is important for firm conclusions to be drawn, we reported that, despite the loss of CD28 expression, these patients surprisingly did not present a broader susceptibility to pathogens other than HPV. In marked contrast, CARMIL2 deficiency confers a predisposition to various bacterial, mycobacterial, and viral pathogens, including HPV, in addition to severe non-infectious complications. The discovery of human CD28 deficiency therefore supports the notion that the susceptibility to skin HPV observed in CARMIL2 deficiency results from impaired CD28-mediated NF-κB activation in T cells. The molecular and cellular function of CARMIL2 remains unclear, but the thorough phenotypic description of humans with CD28 and CARMIL2 deficiencies, and dissection of CD28 signaling in those patients’ cells, has highlighted the existence of a role for CARMIL2 extending beyond CD28 signaling in T cells, as a pleiotropic molecule orchestrating immune responses.

## Materials and methods

### Whole-exome sequencing

We extracted genomic DNA from blood samples collected from the patients with the iPrep PureLink gDNA Blood Kit and iPrep Instruments from Life Technologies. Exome capture was performed with the SureSelect Human All Exon 71 Mb kit (Agilent Technologies). Paired-end sequencing was performed on a HiSeq 2500 machine (Illumina) generating 100-base reads. We aligned the sequences with the GRCh37 reference build of the human genome, using the Burrows-Wheeler aligner. Downstream processing and variant calling were performed with the Genome Analysis Toolkit, SAMtools, and Picard. Substitution and InDel calls were made with the GATK Unified Genotyper. However, various other sequencing approaches were used by our collaborators for the identification of CARMIL2 variants, which were then generally verified by Sanger sequencing.

### Cell culture

PBMCs were isolated by Ficoll-Hypaque density centrifugation (Amersham-Pharmacia-Biotech). PBMCs, Jurkat cells, and PHA and PMA blasts were cultured in RPMI-1640 medium supplemented with 10% FCS.

### Exon trapping

For exon trapping of the c.871G>C allele, a region of genomic DNA encompassing a 3′-fragment of *CARMIL2* intron 10 (120 bp), exon 11 (95 bp), and a 5′-fragment of intron 11 (77 bp) was amplified from the genomic DNA of a HD and inserted into pSPL3 (Life Technologies) between the BamH1 and EcoR1 sites. We obtained the pSPL3 encoding the c.871G>C allele by performing site-directed mutagenesis with the following primers: forward: 5′-GAT​GAC​CGA​CGT​ATG​ACT​GAG​C-3′; and reverse: 5′-GCT​CAG​TCA​TAC​GTC​GGT​CAT​C-3′. The same approach was used for exon trapping of the c.1226 + 1G>T allele, with a pSPL3 plasmid encoding a 984-base-pair fragment of *CARMIL2* genomic DNA extending from intron 12 to intron 16. We obtained the pSPL3 encoding the c.1226 + 1G>T allele by performing site-directed mutagenesis with the following primers: forward: 5′-TCT​CCC​GCA​CTT​AAG​GGG​GAC-3′; and reverse: 5′-GTC​CCC​CTT​AAG​TGC​GGG​AGA-3′. Plasmids encoding the WT, c.871G>C, and c.1226 + 1G>T variants were used to transfect COS7 cells in the presence of X-tremeGENE 9 DNA Transfection Reagent (Roche). Total RNA was extracted with the RNeasy Mini Kit (Qiagen) and used for cDNA synthesis with the SuperScript III First-Strand Synthesis System (Life Technologies). SD6 and SA2 primers were used to amplify spliced transcripts from cDNA specimens by PCR. The PCR product was inserted into the pGEM-T Easy plasmid (Promega). The same approach was used for exon trapping of the c.1128C>T allele, but with genomic DNA encompassing the mutation site amplified from the genomic DNA extracted from the cells of P4. The following primers were used: forward: 5′- GGG​ATC​ACC​AGA​ATT​CCA​ACT​GCT​GAG​TGA​CCC​C-3′; reverse: 5′- CAG​ATA​TCT​GGG​ATC​TCC​GAC​ACT​GAC​CTG​AGC​G-3′.

### Analysis of CARMIL2 expression by FACS

For the evaluation of intracellular CARMIL2 expression by FACS, we first performed extracellular staining of fresh or frozen PBMCs with mAb against CD3 (SP34-2; BD), CD4 (SK3; BD), CD8 (SK1; BD), CD45RA (HI100; Sony) CD27 (L128; BD), and CD19 (SJ25C1; BD) and Aqua Live/Dead Cell Stain Kit (Life Technology) for 30 min at room temperature. The cells were then washed in FACS buffer (1× PBS, 2% FCS, 2 mM EDTA), fixed and permeabilized with a fixation/permeabilization kit (eBioscience; Foxp3/Transcription Factor Fixation/Permeabilization) for 20 min in the dark at room temperature. Cells were washed before intracellular staining with mAb against CARMIL2-PE (EM53; Exbio) or isotype control at a dilution of 1/100 for 1 h at 4°C. Cells were washed three times and analyzed with a Fortessa X20 (BD) cytometer. The data were then analyzed with FlowJo v10 software. The same protocol was used for CARMIL2 expression in Jurkat T cells, after extracellular staining with the Aqua Live/Dead Cell Stain Kit only.

### Phospho-p65 and phospho-SLP76 in Jurkat T cells or PBMCs

Cells were stained by incubation for 10 min at 37°C with the Aqua Live/Dead kit (Thermo Fisher Scientific). We dispensed 10^6^ cells into each well of a 96-well V-bottomed plate and incubated them on ice for 10 min with anti-CD28 (CD28.2; eBioscience) or CD3 (OKT3; eBioscience) mAb, as indicated (5 µg/ml each). Cells were washed twice with cold medium, and a polyclonal goat anti-mouse Ig (BD Biosciences) was added to each well (5 µg/ml) to crosslink activating receptors. PMA (40 ng/ml) was used in separate wells as a positive control for PBMCs. After 20 min of incubation at 37°C, cells were fixed by incubation for 10 min at 37°C with Fix buffer I (BD Biosciences). PBMCs were stained for 30 min with mAb against CD3 (BW264/56; Miltenyi Biotec), CD4 (M-T321; Miltenyi Biotec) and CD8 (BW135/80; Miltenyi Biotec). The cells were then permeabilized by incubation for 20 min at room temperature with Perm buffer III (BD Biosciences) and stained for 3 h at room temperature with anti-NF-κB p65-(pS529)-PE or anti-SLP76-(pY128)-PE Abs (BD Biosciences). Cells were then acquired on a FACS Gallios (Beckman Coulter) flow cytometer and analyzed with FlowJo v10.

### T cell proliferation and IL-2 rescue

Total PBMCs or sorted naive T cells (defined as CD3^+^, CD45RA^+^, CCR7^+^ cells) were stained with carboxy-fluorescein diacetate succinimidyl ester (CFSE, Thermo Fisher Scientific) at a final concentration of 2.5 µM for 5 min at room temperature in the dark. Following two washes to remove residual CFSE, 10^5^ cells were stimulated in a 96-well U-bottom plate with anti-CD3–coupled beads (Bio-anti-CD3, OKT3 from eBioscience coupled with anti-Biotin MACSiBeads from Miltenyi Biotec) at a ratio of 4:1, 500 ng/ml soluble anti-CD28 mAb (CD28.2, eBioscience), or with 0.5 ng/ml PMA (Sigma-Aldrich) and 1 µM ionomycin (Sigma-Aldrich). Titration of the anti-CD3–coupled bead-to-cell ratio had been performed earlier (Magg et al., 2021). For IL-2 (Novartis) rescue experiments, we added 500 IU/ml IL-2. After 4 or 5 d of incubation at 37°C under an atmosphere containing 5% CO_2_, the cells were washed and stained with Abs targeting CD3 (SK7), CD4 (SK3), CD8 (RPA-T8), and CD25 (M-A251, all from BD). Naive T cells were sorted with a BD FACSAria II cell sorter.

### Testing of somatic reversion

We identified the T cell populations in which CARMIL2 reexpression occurred by staining PBMCs with the Abs against the following: CD3 (SK7), CD4 (SK3), CD8 (RPA-T8), CD25 (M-A251, all from BD), CD27 (O323; eBioscience), CD45RA (HI100, BD), CD56 (HCD56; Biolegend), FoxP3 (PCH101; eBioscience), and CARMIL2 (EM53; Exbio). Viability was assessed with Zombie Aqua Live/Dead stain (Biolegend). In patients with CARMIL2-expressing T cell populations, T lymphoblasts were generated by stimulating 10^6^ PBMCs with 5 ng/ml PMA, 1 µM ionomycin, and 100 U/ml IL-2 for 2 d before further expansion for 8–16 d in complete RPMI 1640 supplemented with 100 U/ml IL-2. The T lymphoblasts were sorted with Abs against CD3 (SK7), CD4 (SK3), CD8 (RPA-T8, all from BD), and CARMIL2 (EM53; Exbio). DNA was extracted from the CARMIL2-expressing cell populations with the DNeasy Blood and Tissue kit (Qiagen), and reversion events were confirmed by Sanger sequencing.

### Lentivirus production and transduction

Lentiviruses were produced as follows. 2 d before transduction, 0.5–1.0 × 10^6^ HEK293T cells were used to seed a 6-well plate. The following day, HEK293T cells were transfected with pCMV-VSV-G (0.2 μg), pHXB2-env (0.2 μg; NIH-AIDS Reagent Program; #1069), psPAX2 (1 μg; gift from Didier Trono, Laboratory of Virology and Genetics, School of Life Sciences, Ecole Polytechnique Fédérale de Lausanne, Lausanne, Switzerland; plasmid #12260; Addgene), pTRIP-SFFV-GFP-2A-CARMIL2_isoform 1 (1.6 μg), pTRIP-SFFV-GFP-2A-CARMIL2_isoform 2 (1.6 μg), pTRIP-SFFV-GFP-2A-CARMIL2_isoform 3 (1.6 μg), pTRIP-SFFV-GFP-2A-CARMIL2 plasmids encoding each of the documented alleles in patients, or pTRIP-SFFV-GFP-2A (1.6 μg; empty vector; modified from pTRIP-SFFV-mtagBFP-2A; gift from Nicolas Manel, Institut Curie, PSL Research University, INSERM, U932, Paris, France; plasmid #102585; Addgene) in the presence of X-tremeGENE HP (Sigma-Aldrich), in accordance with the manufacturer’s protocol. The medium was replaced after 8 hours of incubation. In parallel, CARMIL2-deficient Jurkat cells were used to seed 96-well round-bottomed plates at a density of 2 × 10^5^ cells/well. For the transfection of patient cells, T cell blasts derived from a CARMIL2-deficient patient and a healthy control were stimulated with beads coated with Abs against CD2, CD28, and CD3 (T cell activation/expansion kit, Miltenyi Biotec). On day 0, 24 h after the HEK293T cell medium was changed; the viral supernatant was recovered and passed through a filter with 0.2 μm pores. Protamine sulfate (8 μg/ml) was added to the viral supernatant, which was then added to the activated T cells or Jurkat cells (immediately after seeding), which were spinoculated for 2 h at 1,200 *g* and 25°C. After spinoculation, cells were cultured for 48 h at 37°C under an atmosphere containing 5% CO_2_. On day +2, the cells were transferred to a 24-well plate containing RPMI supplemented with 2% human serum AB (Sigma-Aldrich), penicillin/streptomycin (1/1,000) and r-IL2 (10 ng/ml; Thermo Fisher Scientific) for primary T cells, and RPMI supplemented with 10% FCS for Jurkat cells. The medium was replaced on day +5 and the p-p65 experiment was performed on day +6 for primary T cells, or at different time points for Jurkat cells.

### Plasmids, directed mutagenesis, and transient transfection

The pcDNA3.1 plasmid encoding isoform 3 of human CARMIL2 was generated by directed mutagenesis on the pcDNA3.1 encoding isoform 1 (Wang et al., 2016), by deleting a region of 108 nucleotides in exon 14 from position 67,682,033 to position 67,682,140. The pcDNA3.1 plasmid encoding isoform 2 was generated by directed mutagenesis on the pcDNA3.1 encoding isoform 3 by deleting exon 36. The constructs carrying the mutant alleles found in the patients were generated by direct mutagenesis with the CloneAmp HiFi PCR premix kit (Takara). HEK293T cells were transfected with the X-tremeGENE 9 DNA Transfection Reagent (Roche).

### Cell lysis and immunoblotting

Total protein extracts from HEK293T or Jurkat T cells were prepared with lysis buffer (radioimmunoprecipitation assay buffer, aprotinin, dithiothreitol, proteinase inhibitors, PMSF, and leupeptin). Immunoblotting was performed with Abs against the C terminus of CARMIL2 (EM-53; Exbio), N terminus of CARMIL2 (E-15; Santa Cruz), GAPDH (FL335; Santa Cruz), and KU80 (C48E7; Cell Signaling Technologies).

### mRNA analysis and RT-qPCR

Total RNA was extracted with the RNeasy kit (Qiagen), and reverse-transcribed to generate cDNA. qPCR was then performed on the RNA with the Applied Biosystems probes/primers specific for CARMIL2-FAM (HS00418748_m1), IL2-FAM (Hs00174114_m1), NFKB1-FAM (Hs00765730_m1), FOSL2-FAM (Hs01050117_m1), FASLG-FAM (Hs00181226_g1), and 13glucuronidase-VIC (4326320E) for normalization. Results are expressed according to the ΔCt method.

### Deep immunophenotyping by CyTOF

CyTOF was performed on whole blood with the Maxpar Direct Immune Profiling Assay (Fluidigm), according to the supplier’s instructions. Cells were frozen at −80°C after the overnight dead-cell staining step, and acquisition was performed on a Helios machine (Fluidigm). All the samples were processed within 24 h of sampling. Data analysis was performed with OMIQ software.

### scRNA-seq

scRNA-seq datasets were retrieved from our previous study (Ogishi et al., 2021) and from 10x Genomics reference datasets. They were generated from PBMCs of healthy human donors with the Next GEM Single-Cell 3′ GEM Kit v3.1 (10x Genomics) and cDNA libraries prepared and sequenced as previously described (Lévy et al., 2021). Sequence read quality was assessed with BVAtools (https://bitbucket.org/mugqic/bvatools/src/master/), and Cell Ranger v3.0.1 was used for mapping to the hg38 human reference genome assembly, filtering, and counting barcodes and UMIs. We then extracted the summary statistics for the number of genes detected and UMIs per cell and applied our cell quality and doublet-filtering pipeline (Béziat et al., 2021). After the exclusion of dead cells and doublets, the samples were analyzed with the Seurat v4 R package, and cell clustering was performed by the uniform manifold approximation and projection (UMAP) dimension reduction method for all cells together (Becht et al., 2018; Hao et al., 2021). Further reclustering was performed on cells attributed to the T and NK lineages to gain resolution for the different cellular subsets; *CARMIL2* gene expression in each subset was superimposed on the UMAP profile. Published bulk RNA-seq datasets for human untreated naive CD4 T cells and B cells (GSE166872; Lévy et al., 2021), CD8 T cells (GSE107011; Monaco et al., 2019), NK cells (GSE115736; Choi et al., 2019), and untreated pDCs (GSE84204; Alculumbre et al., 2018) were downloaded from GEO with the SRA toolkit (--fastq-dump). The quality of sequence reads was evaluated with FastQC (Babraham Bioinformatics) and low-quality reads and bases were trimmed with Trimmomatic v.0.33 (Bolger et al., 2014). The reads from the biological replicates for each cell type were aligned with the human hg38 assembly, with HISAT2 v2.2.1 (Pertea et al., 2016), to obtain higher coverage for the exon splice junctions. The resulting SAM files were converted to BAM format, sorted, and indexed with samtools v1.12 (Li et al., 2009). The alignments of reads with the CARMIL2 gene were visualized by loading the BAM files into Integrated Genome Viewer (IGV) and spliced reads were counted with the Sashimi plot function (Thorvaldsdóttir et al., 2013).

### RNA-seq

Total RNA was extracted from naive primary CD4^+^ T cells, with the RNeasy Plus Micro Kit (Qiagen). RNA integrity and purity were evaluated with a Bioanalyzer 2100 (Agilent Technologies Genomics). We generated cDNA from the RNA with the SMARTer v4 Ultra Low Input RNA for Sequencing Kit (Takara Bio). The cDNA was quantified, and its size was checked with a Bioanalyzer 2100 (Agilent Technologies Genomics). cDNA concentration was standardized to 1 ng/μl and libraries were prepared with the Nextera XT DNA Library Preparation Kit (Illumina) and the Nextera XT Index Kit v2 set A (Illumina). We performed 150 bp paired-end sequencing to generate ∼20 million reads per sample with a HiSeq4000 system (Illumina). Raw RNA-seq reads were aligned with UCSC human genome assembly version hg38, with STAR aligner (Dobin et al., 2013). We used R version 3.5.2. We normalized the datasets with the functions DGEList and calcNormFactors from the DESeq2 version 1.22.2 package (Love et al., 2014). We retained only genes with more than 10 counts-per-million in at least two samples. We considered a gene to be differentially expressed between two sets of conditions if the log_2_-fold change in expression exceeded 1 (absolute value) and the adjusted P value was below 0.05, according to the calculations made with the DESeq function. The raw and processed RNA-seq data are available from GEO under SuperSeries accession number GSE169506.

### VirScan: Phage immunoprecipitation sequencing

For Ab profiling by phage immunoprecipitation sequencing (Xu et al., 2015), plasma samples from patients and controls (HD) were assayed and data were analyzed as previously described (Drutman et al., 2020; Kerner et al., 2020), but with the following modifications. We calculated species-specific significance cutoff values to estimate the minimum number of enriched, non-homologous peptides required to consider a sample to be seropositive, as previously described with an in-house dataset and a generalized linear model (Xu et al., 2015). For each sample, we calculated virus-specific scores by dividing the counts of enriched, non-homologous peptides by the estimated cutoff score. These adjusted virus scores (Virus Scoreadj) were then visualized on heatmap plots and used for principal component analysis, as shown in Fig. 5 E. Pooled human plasma used for IVIg (Privigen CSL Behring AG) and human IgG-depleted serum (Molecular Innovations, Inc.) served as additional controls. All research on human subjects was performed after informed written consent had been obtained, and procedures were approved by the institutional research ethics boards of Sidra Medicine and Qatar Biobank.

### Study approval

The experiments described here were conducted in accordance with local, national, and international regulations and were approved by the local ethics committee (# 2010-A00634-35; RCB), Agence nationale de sécurité du médicament et des produits de santé (B100711-40), and by the French Ministry of Research (IE-2016-851). Written informed consent for these studies was provided, and ethical/institutional approval was granted by the LMU Munich (19-469). Informed consent was obtained from the patients’ families, for minors, in accordance with World Medical Association rules, the Helsinki Declaration, and European Union directives.

### Online supplemental material

The online supplementary information describes the population genetics of CARMIL2 alleles (Fig. S1), the impact of several variants on *CARMIL2* mRNA splicing, CARMIL2 protein expression (Fig. S1), and CARMIL2 function in terms of phosphor-P65 (Fig. S2), detailed immunophenotyping by CyTOF (Figs. S2 and S3), detailed results on patients’ T cell proliferation and CD25 expression (Fig. S4), results on patients’ with a somatic reversion event (Fig. S5), detailed demographic and genetic information on 89 patients (Table S1), detailed information on in vitro and ex vivo validation of CARMIL2 alleles (Table S2), raw RNA-seq data on sorted and activated T cells (Table S3), Ig levels and serological responses (Table S4), a list of pathogens documented in patients (Table S5), detailed clinical information on EBV^+^ SMTs in patients (Table S6), and uncropped gel pictures corresponding to Western blots shown in Fig. 1 G, Fig. 3 A, and Fig. S1 (source data files).

## Supplementary Material

Table S1 shows cohort of 89 patients with CARMIL2 deficiency.

Table S2 shows in vitro and ex vivo validation of CARMIL2 alleles.

Table S3 shows raw RNA-seq data.

Table S4 shows immunoglobulin levels and serological response to vaccines.

Table S5 is a list of the pathogens documented in CARMIL2-deficient patients.

Table S6 shows characteristics of EBV^+^ SMTs in CARMIL2-deficient patients.

SourceData F1 contains original blots for Fig. 1.

SourceData F3 contains original blots for Fig. 3.

SourceData FS1 contains original blots for Fig. S1.
