# Supplementary material for: Human CARMIL2 deficiency underlies a broader immunological and clinical phenotype than CD28 deficiency
Source: J Exp Med. 2022 Dec 14;220(2):e20220275. doi: 10.1084/jem.20220275 (PMC9754768; doi:10.1084/jem.20220275)
Supplement: Table S1 — shows cohort of 89 patients with CARMIL2 deficiency. [file JEM_20220275_TableS1.docx]

Table S1. Cohort of 89 patients with CARMIL2 deficiency

| Subject no. | Case no. | Sex/age at onset/age at evaluation | Country of origin | CARMIL2 cDNA position; predicted amino-acid change | Exon or intron (Domain) | Type of mutation | CADD score | Reference |
| --- | --- | --- | --- | --- | --- | --- | --- | --- |
| 1 | A.1 | F/2/20 | Portugal | c.1578C>T; NA | 19 (LRR) | Synonymous splicing | NA | . |
| 2 | B.1 | F/1/36 | Pakistan | c.1906_1907del; p.Leu636Alafs*39 | 22 (LRR) | Frameshift | NA | . |
| 3 | B.2 | F/1/35 | Pakistan | c.1906_1907del; p.Leu636Alafs*39 | 22 (LRR) | Frameshift | NA | . |
| 4 | C.1 | F/0.8/11 | Mauritania | c.1128C>T; NA | 14 (LRR) | Synonymous splicing | 15.6 | . |
| 5 | C.2 | F/1/5 | Mauritania | c.1128C>T; NA | 14 (LRR) | Synonymous splicing | 15.6 | . |
| 6 | C.3 | M/UK/10 | Mauritania | c.1128C>T; NA | 14 (LRR) | Synonymous splicing | 15.6 | . |
| 7 | D.1 | M/0.9/6 | Turkey | c.118_119insA; p.Asn41Lysfs*47 | 2 (PH) | Frameshift | NA | . |
| 8 | D.2 | F/0.4/4 | Turkey | c.118_119insA; p.Asn41Lysfs*47 | 2 (PH) | Frameshift | NA | . |
| 9 | D.3 | F/NA*/2 | Turkey | c.118_119insA; p.Asn41Lysfs*47 | 2 (PH) | Frameshift | NA | . |
| 10 | E.1 | F/UK/31 | Tunisia | c.2449C>T; p.Gln817* | 26 (HD) | Nonsense | 39 | Wang et al., 2016 |
| 11 | E.2 | F/UK/29 | Tunisia | c.2449C>T; p.Gln817* | 26 (HD) | Nonsense | 39 | Wang et al., 2016 |
| 12 | F.1 | M/3/10 | Turkey | c.1581-1G>A; NA | Intron 19 (LRR) | Splicing | 23.9 | . |
| 13 | G.1 | M/2/23 | Turkey | c.1466T>A; p.Leu489Gln | 18 (LRR) | Missense | 28 | Wang et al., 2016 |
| 14 | H.1 | F/0.4/15 | Mexico | c.1834delC; p.His612Thrfs*20 | 22 (LRR) | Frameshift | NA | . |
| 15 | I.1 | M/3/16 | USA | c.790C>T; p.Arg264* | 11 (LRR) | Nonsense | 36 | . |
| 16 | J.1 | F/4/6 | Mexico | c.1834delC; p.His612Thrfs*20 | 22 (LRR) | Frameshift | NA | Kim et al., 2019 |
| 17 | K.1 | M/0/22 | Norway | c.1808T>A; p.Leu603His | 21 (LRR) | Missense | 29.9 | Sorte et al., 2016 |
| 18 | L.1 | M/20/56 | Norway | c.1808T>A; p.Leu603His | 21 (LRR) | Missense | 29.9 | Sorte et al., 2016 |
| 19 | M.1 | F/10/37 | Norway | c.1808T>A; p.Leu603His | 21 (LRR) | Missense | 29.9 | Sorte et al., 2016 |
| 20 | M.2 | F/4/42 | Norway | c.1808T>A; p.Leu603His | 21 (LRR) | Missense | 29.9 | Sorte et al., 2016 |
| 21 | N.1 | M/0.2/21 | Morocco | c.1115T>G; p.Leu372Arg | 14 (LRR) | Missense | 27.1 | Wang et al., 2016 |
| 22 | N.2 | F/1/6 | Morocco | c.1115T>G; p.Leu372Arg | 14 (LRR) | Missense | 27.1 | Wang et al., 2016 |
| 23 | N.3 | F/1/6 | Morocco | c.1115T>G; p.Leu372Arg | 14 (LRR) | Missense | 27.1 | Wang et al., 2016 |
| 24 | O.1 | M/1/32 | Saudi Arabia | c.2428-2440del; p.Leu810Serfs*36 | 26 (HD) | Frameshift | NA | Alazami et al., 2018 |
| 25 | O.2 | F/3/16 | Saudi Arabia | c.2428-2440del; p.Leu810Serfs*36 | 26 (HD) | Frameshift | NA | Alazami et al., 2018 |
| 26 | O.3 | M/5/12 | Saudi Arabia | c.2428-2440del; p.Leu810Serfs*36 | 26 (HD) | Frameshift | NA | Alazami et al., 2018 |
| 27 | P.1 | F/9/31 | Saudi Arabia | c.2428-2440del; p.Leu810Serfs*36 | 26 (HD) | Frameshift | NA | Alazami et al., 2018 |
| 28 | P.2 | F/2/37 | Saudi Arabia | c.2428-2440del; p.Leu810Serfs*36 | 26 (HD) | Frameshift | NA | Alazami et al., 2018 |
| 29 | Q.1 | M/0.3/15 | Saudi Arabia | c.149G>C; p.Arg50Thr | 3 (PH) | Missense | 26.8 | Alazami et al., 2018 |
| 30 | Q.2 | M/0/6 | Saudi Arabia | c.149G>C; p.Arg50Thr | 3 (PH) | Missense | 26.8 | Alazami et al., 2018 |
| 31 | R.1 | F/8/25 | Turkey | c.1812-7G>A; NA | Intron 21 (LRR) | Splicing | 11.2 | Kolukisa et al., 2022 |
| 32 | R.2 | F/10/20 | Turkey | c.1812-7G>A; NA | Intron 21 (LRR) | Splicing | 11.2 | Kolukisa et al., 2022 |
| 33 | S.1 | F/22/38 | Turkey | c.311-325del; p.Ala103-Leu107del | 5 (PH) | In-frame deletion | NA | . |
| 34 | T.1 | M/1/12 | Saudi Arabia | c.1256_1285del; p.Gln419_Leu428del | 16 (LRR) | In-frame deletion | NA | Yonkof et al., 2020 |
| 35 | T.2 | M/1/9 | Saudi Arabia | c.1256_1285del; p.Gln419_Leu428del | 16 (LRR) | In-frame deletion | NA | Yonkof et al., 2020 |
| 36 | U.1 | M/0.3/8 | Senegal | c.2635G>T; p.Glu879* | 28 (PRR) | Nonsense | 33.0 | . |
| 37 | V.1 | F/0.5/4.5 | India | c.1226+1G>T; NA | Intron 15 (LRR) | Splicing | 24.6 | . |
| 38 | V.2 | F/0.2/15 | India | c.1226+1G>T; NA | Intron 15 (LRR) | Splicing | 24.6 | . |
| 39 | V.3 | M/10/11 | India | c.1226+1G>T; NA | Intron 15 (LRR) | Splicing | 24.6 | . |
| 40 | W.1 | M/8/23 | India | c.281T>C ; p.Leu94Pro | 5 (PH) | Missense | 25.9 | . |
| 41 | X.1 | M/0.2/9 | Turkey | c.1149+5G>C; NA | Intron 14 (LRR) | Splicing | 9.5 | Kolukisa et al., 2022 |
| 42 | X.2 | M/0.2/5 | Turkey | c.1149+5G>C; NA | Intron 14 (LRR) | Splicing | 9.5 | Kolukisa et al., 2022 |
| 43 | Y.1 | M/0.2/15 | Turkey | c.1149+5G>C; NA | Intron 14 (LRR) | Splicing | 9.5 | Kolukisa et al., 2022 |
| 44 | Y.2 | F/0.2/UK | Turkey | c.1149+5G>C; NA | Intron 14 (LRR) | Splicing | 9.5 | Kolukisa et al., 2022 |
| 45 | AA.1 | F/2/19 | Yemen | c.490dupG; p.Ala164Glyfs*4 | 7 (N-Cap) | Frameshift | NA | Schober et al., 2017 |
| 46 | AA.2 | F2/11 | Yemen | c.490dupG; p.Ala164Glyfs*4 | 7 (N-Cap) | Frameshift | NA | Schober et al., 2017 |
| 47 | BB.1 | M/1/16 | Brazil | c.871+1G>T; NA | Intron 11 (LRR) | Splicing | 25.3 | Schober et al., 2017 |
| 48 | BB.2 | M/1/14 | Brazil | c.871+1G>T; NA | Intron 11 (LRR) | Splicing | 25.3 | Schober et al., 2017 |
| 49 | CC.1 | M/0/17 | Afghanistan | c.958+1G>C; NA | Intron 12 (LRR) | Splicing | 22.5 | . |
| 50 | CC.2 | F/0/7 | Afghanistan | c.958+1G>C; NA | Intron 12(LRR) | Splicing | 22.5 | . |
| 51 | DD.1 | F/1/18 | Kosovo | c.1856T>C; p.Leu619Pro | 22 (LRR) | Missense | 24 | . |
| 52 | EE.1 | M/0.5/37 | Sweden | c.1825G>A; p.Asp609Asn  c.249+1G>T; NA | 22 (LRR)  Intron 4 (PH) | Missense  Splicing | 33  24.8 | . |
| 53 | FF.1 | M/4/32 | Turkey | c.467-1G>A; NA | Intron 6 (LRR) | Splicing | 26.1 | Atschekzei et al., 2019 |
| 54 | FF.2 | F/0.2/9 | Turkey | c.467-1G>A; NA | Intron 6 (LRR) | Splicing | 26.1 | Atschekzei et al., 2019 |
| 55 | FF.3 | M/0.5/5 | Turkey | c.467-1G>A; NA | Intron 6 (LRR) | Splicing | 26.1 | Atschekzei et al., 2019 |
| 56 | GG.1 | M/0.5/6 | Syria | c.1071+1G>T; NA | Intron 13 (LRR) | Splicing | 23.0 | . |
| 57 | HH.1 | F/UK/65 | Germany | c.958+1G>A; NA | Intron 12 (LRR) | Splicing | 23.2 | . |
| 58 | II.1 | F/8/35 | United Kingdom | c.926T>C; p.Leu309Pro  c.1071+2T>A; NA | 12 (LRR)  Intron 13 (LRR) | Missense  Splicing | 25.7  23.3 | . |
| 59 | JJ.1 | F/0.5/15 | Turkey | c.688_689delAG; p.Ser230Profs*2 | 10 (LRR) | Frameshift | NA | Magg et al., 2019 |
| 60 | JJ.2 | M/0.2/4 | Turkey | c.688_689delAG; p.Ser230Profs*2 | 10 (LRR) | Frameshift | NA | Magg et al., 2019 |
| 61 | KK.1 | M/0.5/17 | Russia | c.1974+1_1974+10del; NA | Intron 22 (LRR) | Splicing | NA | Magg et al., 2019 |
| 62 | KK.2 | M/0.5/12 | Russia | c. 1974+1_1974+10del; NA | Intron 22 (LRR) | Splicing | NA | Magg et al., 2019 |
| 63 | LL.1 | M/1.5/33 | Iran | c.1226+1G>T; NA | Intron 15 (LRR) | Splicing | 24.6 | . |
| 64 | MM.1 | F/9/21 | Turkey | c.463delT; p.Cys155Valfs*54 | 6 (LRR) | Frameshift | NA | Kolukisa et al., 2022 |
| 65 | MM.2 | F/4/11 | Turkey | c.463delT; p.Cys155Valfs*54 | 6 (LRR) | Frameshift | NA | Kolukisa et al., 2022 |
| 66 | NN.1 | F/15/17 | Israel | c.691_715delGCCTTGAGGTCTCAGAACAGATTCT; p.Leu231Thrfs*2 | 11 (LRR) | Frameshift | NA | Shamriz et al., 2020 |
| 67 | NN.2 | F/1/9 | Israel | c.691_715delGCCTTGAGGTCTCAGAACAGATTCT; p.Leu231Thrfs*2 | 11 (LRR) | Frameshift | NA | Shamriz et al., 2020 |
| 68 | NN.3 | F/10/15 | Israel | c.691_715delGCCTTGAGGTCTCAGAACAGATTCT; p.Leu231Thrfs*2 | 11 (LRR) | Frameshift | NA | Shamriz et al., 2020 |
| 69 | NN.4 | F/2/9 | Israel | c.691_715delGCCTTGAGGTCTCAGAACAGATTCT; p.Leu231Thrfs*2 | 11 (LRR) | Frameshift | NA | Shamriz et al., 2020 |
| 70 | OO.1 | F/0.5/8 | Pakistan | c.871G>C | 11 (LRR) | Splicing | 28.8 | . |
| 71 | OO.2 | M/0.3/4 | Pakistan | c.871G>C | 11 (LRR) | Splicing | 28.8 | . |
| 72 | PP.1 | F/2/9 | Israel | c.1865C>T; p.Ala622Val | 22 (LRR) | Missense | 34 | . |
| 73 | PP.2 | M/UK/36 | Israel | c.1865C>T; p.Ala622Val | 22 (LRR) | Missense | 34 | . |
| 74 | QQ.1 | M/3/12 | Israel | c.2374C>T; p.Gln792* | 26 (HD) | Nonsense | 38 | . |
| 75 | RR.1 | M/5/UK | India | c.1544_1545delAT; p.His515Argfs*40 | 19 (LRR) | Frameshift | NA | Magg et al., 2019 |
| 76 | SS.1 | M/0.5/14 | Israel | c.1482C>A; p.Asn494Lys | 16 (LRR) | Missense | NA | Kurolap et al., 2019 |
| 77 | TT.1 | F/14/18 | Russia | c.1559_1562delGGAA; p.Arg520Thrfs*38  c.1622T>G; p.Met541Arg | 19 (LRR)  20 (LRR) | Frameshift  Missense | NA  23.6 | . |
| 78 | UU.1 | M/0.5/17 | Germany | c.887_897delinsTGTTGTCCTG; p.Ser296Metfs*10  c.1874T>C; p.Leu625Pro | 12 (LRR)  22 (LRR) | Frameshift  Missense | NA  28.6 | . |
| 79 | VV.1 | F/0.5/16 | Turkey | c.1149+5G>C; NA | 14 (LRR) | Splicing | NA | Kolukisa et al., 2022 |
| 80 | VV.2 | M/2/4 | Turkey | c.1149+5G>C; NA | 14 (LRR) | Splicing | NA | Kolukisa et al., 2022 |
| 81 | VV.3 | F/0.1/10 | Turkey | c.1149+5G>C; NA | 14 (LRR) | Splicing | NA | Kolukisa et al., 2022 |
| 82 | WW.1 | F/2/16 | Turkey | c.902delG; p.Arg301Leufs*6 | 12 (LRR) | Frameshift | NA | Kolukisa et al., 2022 |
| 83 | XX.1 | F/0/18 | Pakistan | c.611+5G>A; NA | Intron 8 (LRR) | Splicing | NA | . |
| 84 | XX.2 | F/0/9 | Pakistan | c.611+5G>A; NA | Intron 8 (LRR) | Splicing | NA | . |
| 85 | YY.1 | M/1/6 | Turkey | c.1071+1G>T; NA | Intron 13 (LRR) | Splicing | 23.0 | . |
| 86 | YY.2 | M/0.3/12 | Turkey | c.1071+1G>T; NA | Intron 13 (LRR) | Splicing | 23.0 | . |
| 87 | ZZ.1 | M/2/21 | Turkey | c.959-2A>T | Intron 12(LRR) | Splicing | 22.8 | . |
| 88 | ZZ.2 | F/2/14 | Turkey | c.959-2A>T | Intron 12(LRR) | Splicing | 22.8 | . |
| 89 | AB.1 | M/5/45 | Germany | c.1109C>A | 14 (LRR) | Nonsense | 29.9 | . |

(*) Subject no. 9 received pre-emptive HSCT, before any symptoms occurred.

UK, unknown; NA, not applicable.

**References**

Alazami, A.M., M. Al-Helale, S. Alhissi, B. Al-Saud, H. Alajlan, D. Monies, Z. Shah, M. Abouelhoda, R. Arnaout, H. Al-Dhekri, et al. 2018. Novel CARMIL2 Mutations in Patients with Variable Clinical Dermatitis, Infections, and Combined Immunodeficiency. *Front. Immunol.* 9:203. https://doi.org/10.3389/fimmu.2018.00203

Atschekzei, F., R. Jacobs, M. Wetzke, G. Sogkas, C. Schröder, G. Ahrenstorf, A. Dhingra, H. Ott, U. Baumann, and R.E. Schmidt. 2019. A Novel CARMIL2 Mutation Resulting in Combined Immunodeficiency Manifesting with Dermatitis, Fungal, and Viral Skin Infections As Well as Selective Antibody Deficiency. *J. Clin. Immunol.* 39:274–276. https://doi.org/10.1007/s10875-019-00628-1

Kim, D., A. Uner, A. Saglam, A. Chadburn, and G.M. Crane. 2019. Peripheral eosinophilia in primary immunodeficiencies of actin dysregulation: A case series of Wiskott-Aldrich syndrome, CARMIL2 and DOCK8 deficiency and review of the literature. *Ann. Diagn. Pathol.* 43:151413. https://doi.org/10.1016/j.anndiagpath.2019.151413

Kolukisa, B., D. Baser, B. Akcam, J. Danielson, S. Bilgic Eltan, Y. Haliloglu, A.P. Sefer, R. Babayeva, G. Akgun, L.-M. Charbonnier, et al. 2022. Evolution and long-term outcomes of combined immunodeficiency due to CARMIL2 deficiency. *Allergy*. 77:1004–1019. https://doi.org/10.1111/all.15010

Kurolap, A., O. Eshach Adiv, L. Konnikova, L. Werner, C. Gonzaga-Jauregui, M. Steinberg, V. Mitsialis, A. Mory, M.Y. Nunberg, S. Wall, et al. 2019. A Unique Presentation of Infantile-Onset Colitis and Eosinophilic Disease without Recurrent Infections Resulting from a Novel Homozygous CARMIL2 Variant. *J. Clin. Immunol.* 39:430–439. https://doi.org/10.1007/s10875-019-00631-6

Magg, T., A. Shcherbina, D. Arslan, M.M. Desai, S. Wall, V. Mitsialis, R. Conca, E. Unal, N. Karacabey, A. Mukhina, et al. 2019. CARMIL2 Deficiency Presenting as Very Early Onset Inflammatory Bowel Disease. *Inflamm. Bowel Dis.* 25:1788–1795. https://doi.org/10.1093/ibd/izz103

Schober, T., T. Magg, M. Laschinger, M. Rohlfs, N.D. Linhares, J. Puchalka, T. Weisser, K. Fehlner, J. Mautner, C. Walz, et al. 2017. A human immunodeficiency syndrome caused by mutations in CARMIL2. *Nat. Commun.* 8:14209. https://doi.org/10.1038/ncomms14209

Shamriz, O., A.J. Simon, A. Lev, O. Megged, O. Ledder, E. Picard, L. Joseph, V. Molho-Pessach, Y. Tal, P. Millman, et al. 2020. Exogenous interleukin-2 can rescue in-vitro T cell activation and proliferation in patients with a novel capping protein regulator and myosin 1 linker 2 mutation. *Clin. Exp. Immunol.* 200:215–227. https://doi.org/10.1111/cei.13432

Sorte, H.S., L.T. Osnes, B. Fevang, P. Aukrust, H.C. Erichsen, P.H. Backe, T.G. Abrahamsen, O.B. Kittang, T. Øverland, S.N. Jhangiani, et al. 2016. A potential founder variant in *CARMIL2/RLTPR* in three Norwegian families with warts, molluscum contagiosum, and T-cell dysfunction. *Mol. Genet. Genomic Med.* 4:604–616. https://doi.org/10.1002/mgg3.237

Wang, Y., C.S. Ma, Y. Ling, A. Bousfiha, Y. Camcioglu, S. Jacquot, K. Payne, E. Crestani, R. Roncagalli, A. Belkadi, et al. 2016. Dual T cell- and B cell-intrinsic deficiency in humans with biallelic RLTPR mutations. *J. Exp. Med.* 213:2413–2435. https://doi.org/10.1084/jem.20160576

Yonkof, J.R., A. Gupta, C.M. Rueda, S. Mangray, B.T. Prince, H.G. Rangarajan, M. Alshahrani, E. Varga, T.P. Cripe, and R.S. Abraham. 2020. A Novel Pathogenic Variant in *CARMIL2* ( *RLTPR*) Causing CARMIL2 Deficiency and EBV-Associated Smooth Muscle Tumors. *Front. Immunol.* 11:884. https://doi.org/10.3389/fimmu.2020.00884
