# Supplementary material for: Human CARMIL2 deficiency underlies a broader immunological and clinical phenotype than CD28 deficiency
Source: J Exp Med. 2022 Dec 14;220(2):e20220275. doi: 10.1084/jem.20220275 (PMC9754768; doi:10.1084/jem.20220275)
Supplement: Table S2 — shows in vitro and ex vivo validation of CARMIL2 alleles. [file JEM_20220275_TableS2.docx]

Table S2. In vitro and ex vivo validation of CARMIL2 alleles

| CARMIL2 cDNA position | Predicted amino acid change | CARMIL2 expression | | Functional validation | | | References |
| --- | --- | --- | --- | --- | --- | --- | --- |
|  |  | Transduced Jurkat T cells | Endogenous | Impact on splicing (exon trapping or TA cloning) | Phospho-P65  (Jurkat T cells) | Primary cells |  |
| c.118_119insA | p.Asn41Lysfs*47 |  | Absent (FC) |  |  | Phospho-P65 |  |
| c.149G>C; | p.Arg50Thr | **Reduced** | Reduced (FC) |  | **Reduced** | Proliferation |  |
| c.249+1G>T | NA |  | Absent (FC)^§^ |  |  | Proliferation |  |
| c.281T>C | p.Leu94Pro | **Reduced** | NT |  | **Reduced** | NT |  |
| c.311_325del | p.Ala103-Leu107del | **Reduced** | Reduced (FC) |  | **Null** | NT |  |
| c.334_337delGCTG | p.Ala112Glnfs*96 |  | Reduced (FC)^§^ |  |  | NT |  |
| c.463delT | p.Cys155Valfs*54 |  | Reduced (FC) |  |  | NT |  |
| c.467-1G>A | NA |  | Absent (FC)^#^ |  |  | Phospho-P65; Proliferation | Atschekzei et al., 2019 |
| c.490dupG | p.Ala164Glyfs*4 |  | Absent (WB) |  |  | Proliferation | Schober et al., 2017 |
| c.611+5G>A | NA |  | Reduced (FC) |  |  | Proliferation; |  |
| c.688_689delAG | p.Ser230Profs*2 |  | Absent (WB) |  |  | Proliferation | Magg et al., 2019 |
| c.691_715delGCCTTGAGGTCTCAGAACAGATTCT | p.Leu231Thrfs*2 |  | Absent (FC) |  |  | Proliferation |  |
| c.790C>T | p.Arg264* |  | Absent (FC) |  |  | NT |  |
| c.871G>C | NA |  | Absent (FC) | Abolishes donor site of exon 11  (Exon trapping) |  | Proliferation |  |
| c.871+1G>T | NA |  | Absent (WB) |  |  | Proliferation | Schober et al., 2017 |
| c.887_897delinsTGTTGTCCTG | p.Ser296Metfs*10 |  | Reduced (FC)^§^ |  |  | Proliferation |  |
| c.902delG | p.Arg301Leufs*6 |  | Reduced (FC) |  |  | NT | Kolukisa et al., 2022 |
| c.926T>C | p.Leu309Pro | **Reduced** | Absent (FC) ^§^ |  | **Null** | Proliferation |  |
| c.958+1G>A | NA |  | Reduced (FC)^#^ |  |  | Proliferation |  |
| c.958+1G>C | NA |  | Reduced (FC) |  |  | Proliferation |  |
| c.959-2A>T | NA |  | Reduced (FC) |  |  | Phospho-P65 |  |
| c.1071+1G>T | NA |  | Absent (FC) |  |  | Phospho-P65; Proliferation |  |
| c.1071+2T>A | NA |  | Absent (FC)^§^ |  |  | Proliferation |  |
| c.1109C>A | p.Ser370* |  | Reduced (FC)^#^ |  |  | Proliferation |  |
| c.1115T>G | p.Leu372Arg | **Reduced** | Absent (FC) |  | **Null** | Phospho-P65; Proliferation |  |
| c.1128C>T | Synonymous |  | Absent (FC) | Novel donor site in exon 14 (c.1127_1449del; p.Gly376Alafs*111)  (exon trapping) |  |  |  |
| c.1149+5G>C | NA |  | Absent (FC) |  |  | NT | Kolukisa et al., 2022 |
| c.1226+1G>T | NA |  | NT | Abolishes donor site of exon 15  (Exon trapping) |  | NT |  |
| c.1256_1285del | p.Gln419_Leu428del | **Reduced** | Reduced (FC) |  | **Null** | Proliferation |  |
| c.1466T>A | p.Leu489Gln | **Reduced** | Absent (FC) |  | **Null** | Phospho-P65; Proliferation |  |
| c.1482C>A | p.Asn494Lys | **Reduced** | Reduced (WB) |  | **Null** | Proliferation | Kurolap et al., 2019 |
| c.1544_1545delAT | p.His515Argfs*40 |  | Absent (WB) |  |  | NT | Magg et al., 2019 |
| c.1559_1562delGGAA | p.Arg520Thrfs*38 |  | NT |  |  | NT |  |
| c.1578C>T | Synonymous |  | Absent (FC)^#^ | (c.1577_1580del;p.C526Wfs*32)  (TA cloning) |  | NT |  |
| c.1581-1G>A | NA |  | Reduced (FC) |  |  |  |  |
| c.1622T>G | p.Met541Arg | **Reduced** | NT |  | **Null** | NT |  |
| c.1808T>A | p.Leu603His | **Reduced** | Reduced (FC) |  | **Null** | NT |  |
| c.1812-7G>A | NA |  | Absent (FC) | In-frame retention of intron 21; c.1812_1869del (p.S605Rfs*8); c.1812_1974del (exon 22 deletion)  (TA cloning) |  | NT |  |
| c.1825G>A | p.Asp609Asn | **Reduced** | Absent (FC)^§^ |  | **Null** | Proliferation |  |
| c.1834delC | p.His612Thrfs*20 |  | NT |  |  | NT |  |
| c.1856T>C | p.Leu619Pro | **Reduced** | Reduced (FC) |  | **Null** | Proliferation |  |
| c.1865C>T | p.Ala622Val | **Reduced** | NT | Neutral effect on splicing | **Null** | NT |  |
| c.1874T>C | p.Leu625Pro | **Reduced** | Reduced (FC) ^§^ |  | **Null** | Proliferation |  |
| c.1906_1907del | p.Leu636Alafs*39 |  | Absent (FC) |  |  | NT |  |
| c.1974+1_1974+10del | NA |  | Absent (WB) |  |  | Proliferation | Magg et al., 2019 |
| c.2374C>T | p.Gln792* |  | NT |  |  | NT |  |
| c.2428_2440del | p.Leu810Serfs*36 |  | Reduced (FC) |  |  | Proliferation |  |
| c.2449C>T | p.Gln817* |  | Absent (FC) |  |  | Phospho-P65; Proliferation |  |
| c.2635G>T | p.Glu879* |  | Absent (FC) |  |  | NT |  |

§ tested only as part of a compound-heterozygous variant in primary patient cells.

# Partial reversion detected

The reference citations refer to validation experiments performed by collaborators. FC, flow cytometry; NT, not tested; WB, Western blot.

**References**

Atschekzei, F., R. Jacobs, M. Wetzke, G. Sogkas, C. Schröder, G. Ahrenstorf, A. Dhingra, H. Ott, U. Baumann, and R.E. Schmidt. 2019. A Novel CARMIL2 Mutation Resulting in Combined Immunodeficiency Manifesting with Dermatitis, Fungal, and Viral Skin Infections As Well as Selective Antibody Deficiency. *J. Clin. Immunol.* 39:274–276. https://doi.org/10.1007/s10875-019-00628-1

Kolukisa, B., D. Baser, B. Akcam, J. Danielson, S. Bilgic Eltan, Y. Haliloglu, A.P. Sefer, R. Babayeva, G. Akgun, L.-M. Charbonnier, et al. 2022. Evolution and long-term outcomes of combined immunodeficiency due to CARMIL2 deficiency. *Allergy*. 77:1004–1019. https://doi.org/10.1111/all.15010

Kurolap, A., O. Eshach Adiv, L. Konnikova, L. Werner, C. Gonzaga-Jauregui, M. Steinberg, V. Mitsialis, A. Mory, M.Y. Nunberg, S. Wall, et al. 2019. A Unique Presentation of Infantile-Onset Colitis and Eosinophilic Disease without Recurrent Infections Resulting from a Novel Homozygous CARMIL2 Variant. *J. Clin. Immunol.* 39:430–439. https://doi.org/10.1007/s10875-019-00631-6

Magg, T., A. Shcherbina, D. Arslan, M.M. Desai, S. Wall, V. Mitsialis, R. Conca, E. Unal, N. Karacabey, A. Mukhina, et al. 2019. CARMIL2 Deficiency Presenting as Very Early Onset Inflammatory Bowel Disease. *Inflamm. Bowel Dis.* 25:1788–1795. https://doi.org/10.1093/ibd/izz103

Schober, T., T. Magg, M. Laschinger, M. Rohlfs, N.D. Linhares, J. Puchalka, T. Weisser, K. Fehlner, J. Mautner, C. Walz, et al. 2017. A human immunodeficiency syndrome caused by mutations in CARMIL2. *Nat. Commun.* 8:14209. https://doi.org/10.1038/ncomms14209
