# Supplementary material for: Human CARMIL2 deficiency underlies a broader immunological and clinical phenotype than CD28 deficiency
Source: J Exp Med. 2022 Dec 14;220(2):e20220275. doi: 10.1084/jem.20220275 (PMC9754768; doi:10.1084/jem.20220275)
Supplement: Table S4 — shows immunoglobulin levels and serological response to vaccines. [file JEM_20220275_TableS4.docx]

Table S4. Immunoglobulin levels and serological response to vaccines

| Immunoglobulins | | | | |
| --- | --- | --- | --- | --- |
|  | *N* | High | Normal | Low |
| IgM | 79 | 23 (29%) | 50 (63%) | 6 (8%) |
| IgG | 80 | 5 (6%) | 63 (79%) | 12 (15%) |
| IgA | 71 | 23 (32%) | 44 (62%) | 4 (6%) |
| IgE | 48 | 12 (25%) | 36 (75%) | 0 (0%) |
|  |  |  |  |  |
| Specific antibodies against booster vaccines | | | | |
|  | *N* |  | Normal | Low |
| Tetanus | 32 |  | 9 (28%) | 23 (72%) |
| Diphtheria | 15 |  | 0 (0%) | 15 (100%) |
| Pneumococcus | 25 |  | 16 (64%) | 9 (36%) |
