# Supplementary material for: Human CARMIL2 deficiency underlies a broader immunological and clinical phenotype than CD28 deficiency
Source: J Exp Med. 2022 Dec 14;220(2):e20220275. doi: 10.1084/jem.20220275 (PMC9754768; doi:10.1084/jem.20220275)
Supplement: Table S5 — is a list of the pathogens documented in CARMIL2-deficient patients. [file JEM_20220275_TableS5.docx]

Table S5. List of the pathogens documented in CARMIL2-deficient patients

|  | Name | No. of patients |
| --- | --- | --- |
| Viruses | Epstein Barr virus | 37 |
|  | Molluscum contagiosum virus | 36 |
|  | Human papillomavirus | 28 |
|  | Cytomegalovirus | 21 |
|  | Varicella zoster virus | 6 |
|  | Herpes simplex virus 1/2 | 5 |
|  | BK virus | 2 |
|  | Norovirus | 1 |
| Bacteria | *Staphylococcus aureus* | 24 |
|  | *Haemophilus influenzae* | 8 |
|  | *Streptococcus pneumoniae* | 6 |
|  | *Pseudomonas aeruginosa* | 2 |
|  | *Mycoplasma pneumoniae* | 2 |
|  | *Salmonella* sp. | 1 |
|  | *Neisseria* sp. | 1 |
|  | *Klebsiella* sp. | 1 |
|  | *Nocardia* sp. | 1 |
| Mycobacteria | *M. avium* | 3 |
|  | *M. tuberculosis* | 2 |
|  | *M. chelonae* | 1 |
| Fungi | *Candida albicans* | 21 |
|  | *Aspergillus niger* | 1 |
|  | *Trychophyton* sp. | 1 |
| Parasites | *Gardia* sp. | 1 |
|  | *Leishmania* sp. | 1 |
