# Supplementary material for: Human CARMIL2 deficiency underlies a broader immunological and clinical phenotype than CD28 deficiency
Source: J Exp Med. 2022 Dec 14;220(2):e20220275. doi: 10.1084/jem.20220275 (PMC9754768; doi:10.1084/jem.20220275)
Supplement: Table S6 — shows characteristics of EBV+ SMTs in CARMIL2-deficient patients. [file JEM_20220275_TableS6.docx]

Table S6. Characteristics of EBV^+^ SMTs in CARMIL2-deficient patients

| Patient no. | Locations | Age at SMT diagnosis | Number in total | EBV-positive | Treatment | Alive | Cause of death |
| --- | --- | --- | --- | --- | --- | --- | --- |
| 1 | Lung | 13 | 1 | 1 | Surgery | Yes |  |
| 16 | Intestine, liver, spine | 4 | Several | 1 | EBV-specific CTLs, pembrolizumab | Yes |  |
| 19 | Scapula | 24 | 1 | UK | Surgery, radiation | Yes |  |
| 34 | Skull, spine, colon, gallbladder, kidneys, lung, duodenum, pancreas | 11 | Multiple | 1 | HSCT | No | SMT progression |
| 35 | Lung, liver, spleen | 8 | 17 | 1 | HSCT | Yes |  |
| 41 | Adrenal gland | 8 | 2 | UK | HSCT | Yes |  |
| 42 | Adrenal gland | 3 | 2 | 1 | HSCT | Yes |  |
| 45 | Gut | 7 | Several | 1 | HSCT planned | Yes |  |
| 46 | Gut, liver, spleen, kidney, brain | 4 | 30 | 1 | Cyclophosphamide | No | SMT progression |
| 47 | Gut, liver, lung | 9 | Multiple | 1 | MTX, vinblastine | No | SMT progression |
| 49 | Gut, liver | 6 | Multiple | 1 | MTX, vinblastine | No | SMT progression |
| 50 | Sigmoideum | 16 | 2 | 1 | HSCT | Yes |  |
| 56 | Liver | 6 | 2 | 1 | Surgery | Yes |  |
| 62 | Adrenal gland, liver | 8 | 2 | 1 | HSCT planned | Yes |  |
| 77 | Adrenal glands | 17 | 4 | 1 | Surgery | No | Complication of surgery |
