# Supplementary figures and images for: Human CARMIL2 deficiency underlies a broader immunological and clinical phenotype than CD28 deficiency
Source: J Exp Med. 2022 Dec 14;220(2):e20220275. doi: 10.1084/jem.20220275 (PMC9754768; doi:10.1084/jem.20220275)

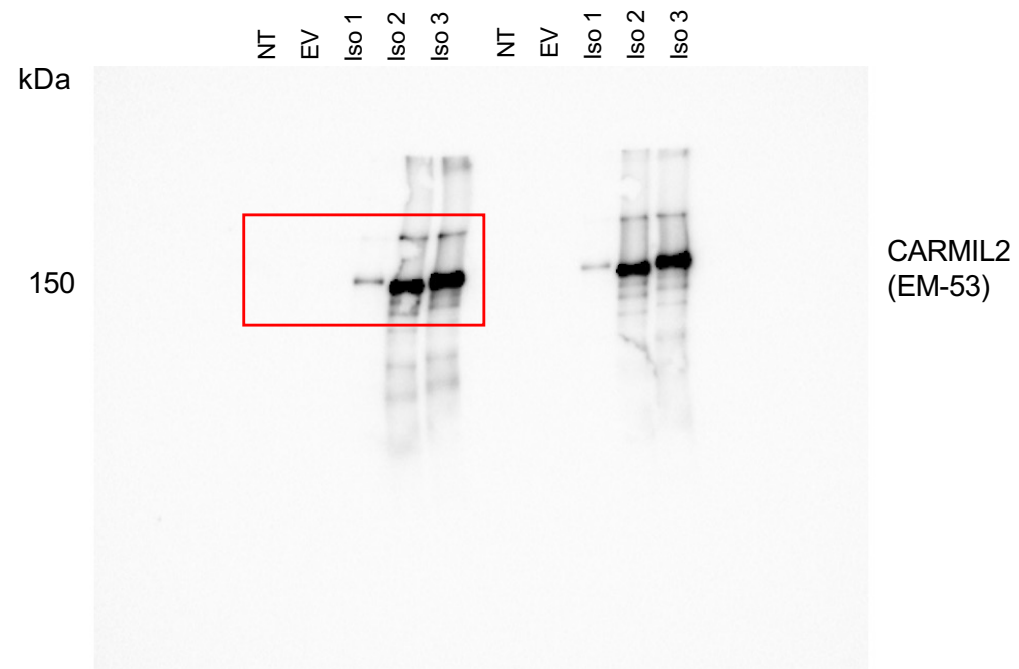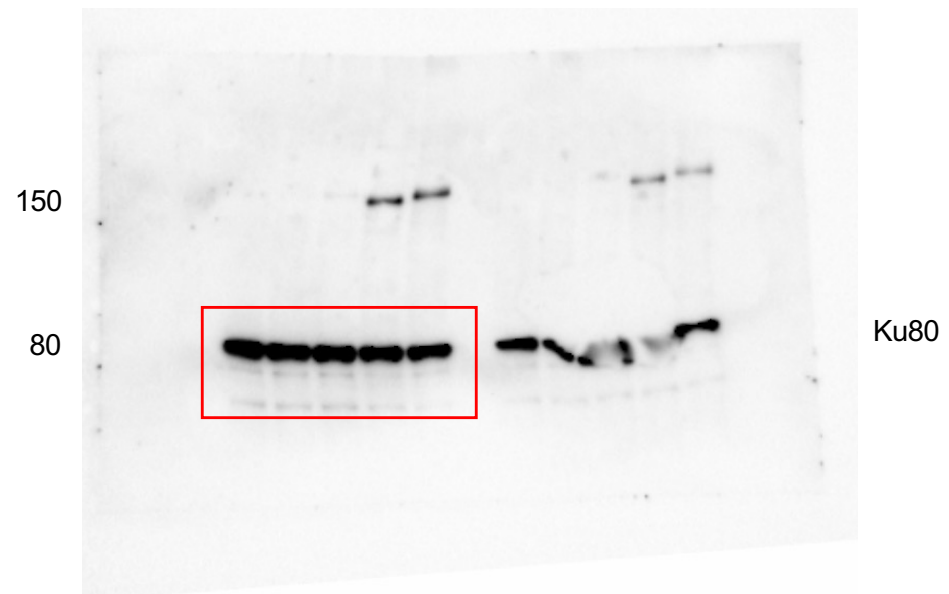

Supplement: SourceData F1 — contains original blots for Fig. 1. [file JEM_20220275_SourceDataF1.pdf]

# HEK293T

Isoform 3

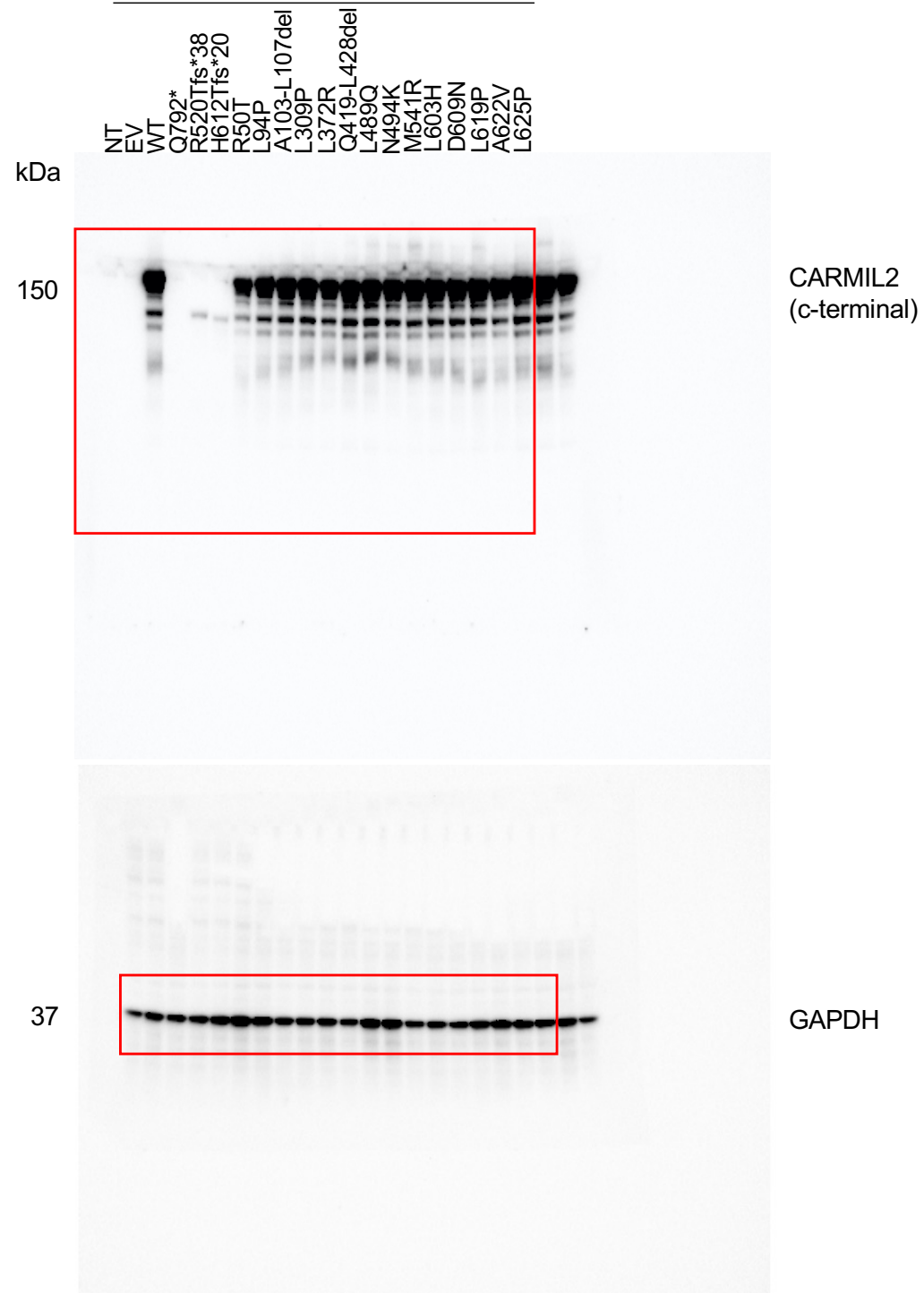

Supplement: SourceData F3 — contains original blots for Fig. 3. [file JEM_20220275_SourceDataF3.pdf]

1H

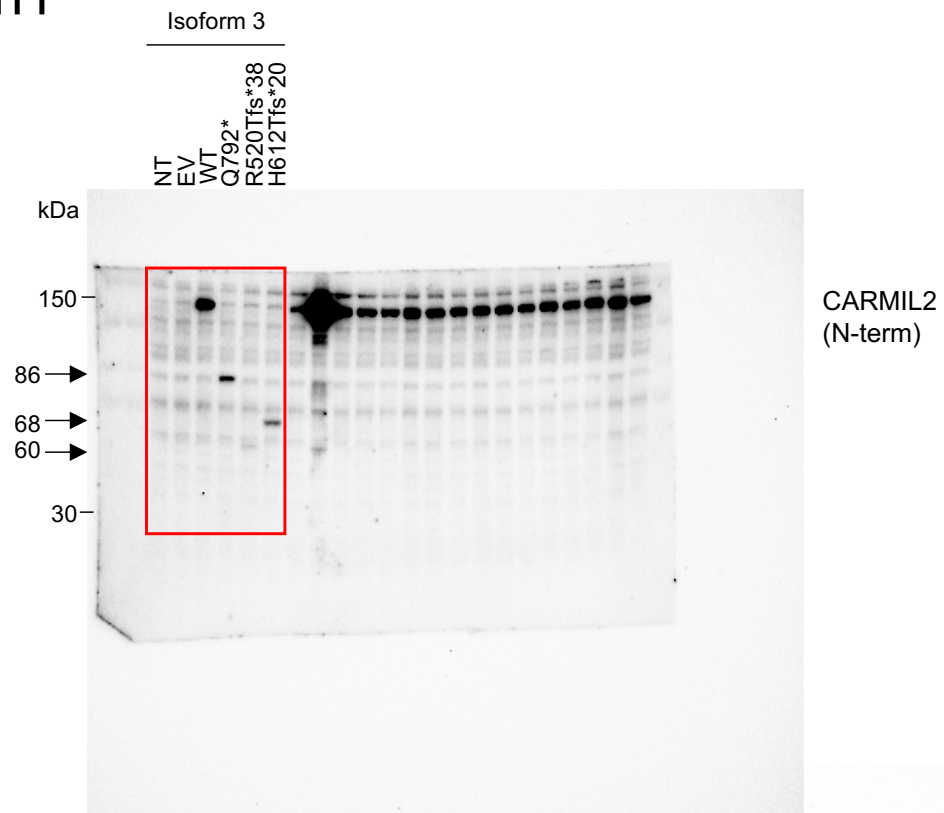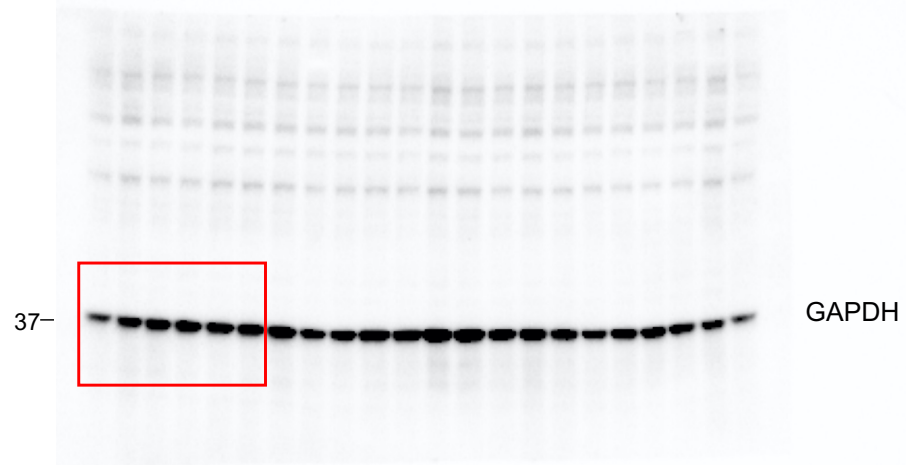

1I

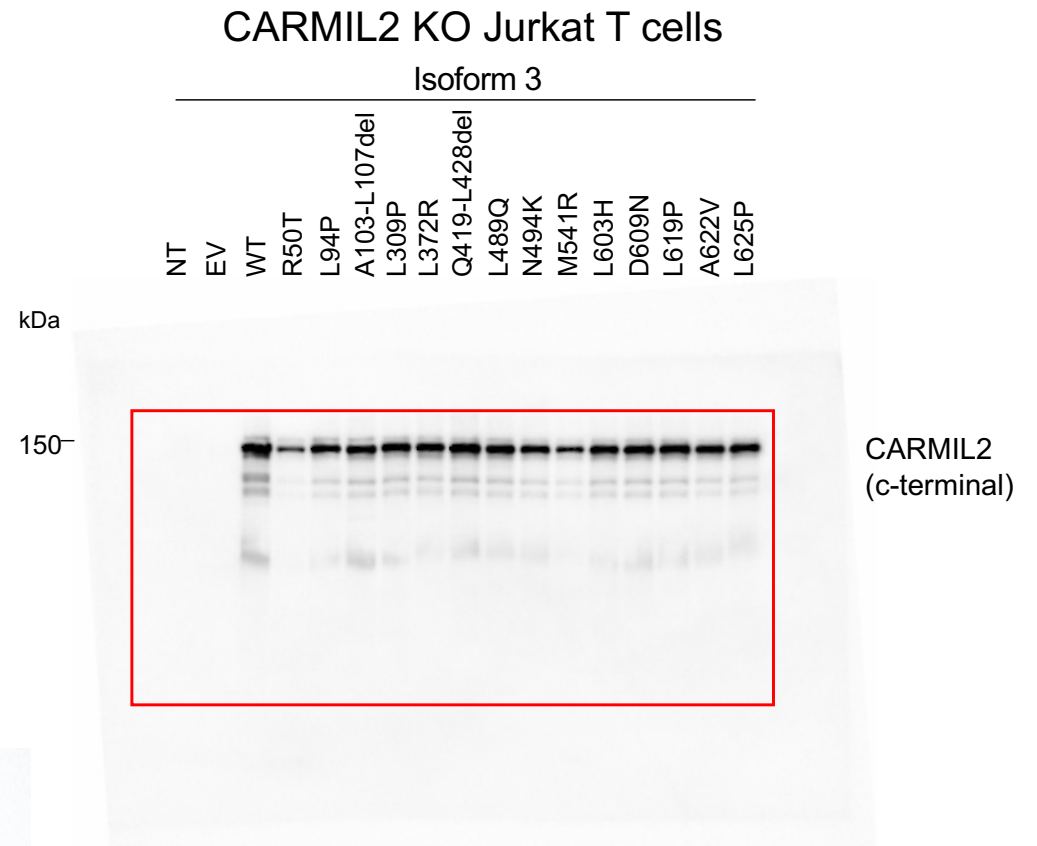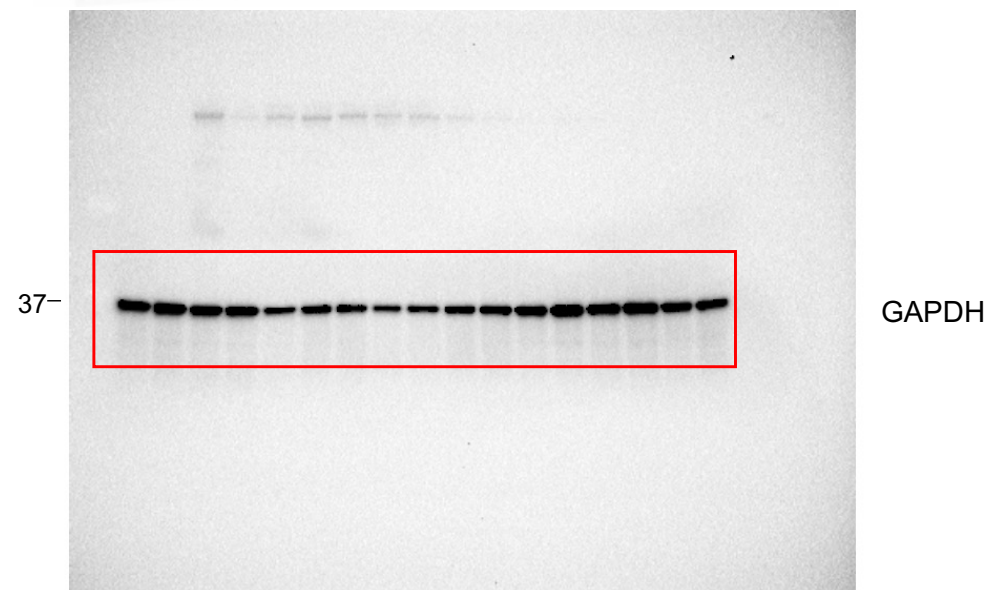

Supplement: SourceData FS1 — contains original blots for Fig. S1. [file JEM_20220275_SourceDataFS1.pdf]
